# Predicting progression from amnestic mild cognitive impairment to Alzheimer's disease using longitudinal EEG data: a 12-month cohort study

**DOI:** 10.3389/fnagi.2025.1719981

**Published:** 2026-01-20

**Authors:** Yingfeng Ge, Yi Fei, Chonglong Ding, Shuo Yang, Yingying Fang, Yifan Zheng, Jianan Yin, Qi Pan, Nanxiang Zhang, Xiaohao Zhang, Xilin Lu, Jinxin Zhang

**Affiliations:** 1 Department of Medical Statistics, School of Public Health, Sun Yat-sen University, Guangzhou, China; 2 Department of Neurology, The First Affiliated Hospital, Sun Yat-sen University, Guangzhou, China; 3 Department of Quality Management, The Third People's Hospital of Yuhang District, Hangzhou, China

**Keywords:** amnestic mild cognitive impairment, EEG, prediction, machine learning, longitudinal data, cohort study

## Abstract

**Introduction:**

Amnestic mild cognitive impairment (aMCI), serving as a clinical precursor to Alzheimer's Disease (AD), assumes a pivotal role in the early stages of AD prevention. The longitudinal collection of data in aMCI is imperative for monitoring disease progression and guiding clinical interventions.

**Methods:**

Utilizing a prospective cohort design, we recruited aMCI individuals and conducted a one-year follow-up study. During this period, electroencephalogram (EEG) signals were systematically collected at regular intervals, resulting in four time points for each participant. Based on the follow-up outcomes, participants were stratified into progressive mild cognitive impairment (PMCI) and stable mild cognitive impairment (SMCI) groups. We extracted spectral, nonlinear, and functional connectivity features from the EEG data at three cross-sectional time points in the initial nine months and constructed longitudinal features between these cross-sectional assessments. The longitudinal features were fed into machine learning classifiers to predict one-year follow-up outcomes.

**Results:**

The dynamic trends of EEG features in SMCI and PMCI patients exhibited inconsistency. Utilizing the selected longitudinal features, the support vector machine (SVM) demonstrated the best prediction performance, achieving an accuracy of 94.92%, an area under the curve of 93.25%, a sensitivity of 90.20%, a specificity of 98.80%, a positive predictive value of 98.70%, and an F1-score of 93.65%.

**Discussion:**

By capturing trend information associated with disease progression, longitudinal EEG features contributed to enhancing prediction performance in machine learning models.

## 1 Introduction

AD, characterized by irreversible memory impairment, aphasia, apraxia, agnosia, and changes in personality and behavioral patterns (Ossenkoppele et al., 2015), onsets insidiously with a prolonged course. Currently, more than 50 million people worldwide are afflicted with AD (Weidner and Barbarino, 2019). Lacking effective medications, this number is expected to escalate to 131 million by 2050, imposing a substantial burden on both society and families (Winblad et al., 2016). Mild Cognitive Impairment (MCI) is a transitional stage that falls between normal age-related cognitive decline and dementia (particularly AD), representing the earliest clinically detectable stage of progression toward dementia or AD (Markesbery, 2010). AMCI, identified with memory dysfunction, is a subtype of MCI with an annual progression rate to AD ranging from 10% to 15% (Cai et al., 2020) and a lifetime conversion rate ranging from 75% to 80% (Gómez-Soria et al., 2021). Therefore, the early and accurate prediction of progression in the aMCI stage becomes a crucial issue in managing the AD continuum and alleviating its burden.

The diagnosis of MCI requires a combination of comprehensive examinations, including biomarkers, neuroimaging, cognitive tests, and neuropsychological assessments (Alzheimer's disease facts figures, 2023). This process is time-consuming, labor-intensive, and cost-prohibitive. Moreover, the insidious onset can be easily misinterpreted as age-related cognitive decline, thus significantly reducing the detection rate of MCI in clinical practice. As a non-invasive examination, EEG presents the benefits of convenience, cost-effectiveness, real-time diagnosis, and wide accessibility. It has been universally applied for the diagnosis and monitoring of disease progression in MCI (Rossini et al., 2022). Compared to task-related EEG, resting-state EEG does not require examinees to perform complex instructions and actions, making it particularly suitable for elders with cognitive decline. Currently, researchers have investigated the EEG signal characteristics in MCI populations, revealing a tendency of “high to low” frequency shift, reduced complexity, and a disconnection phenomenon (Movahed and Rezaeian, 2022). However, the heterogeneity in the disease progression of MCI individuals is also manifested in EEG signals (Libon et al., 2010), presenting substantial challenges for researchers in EEG analysis (Ding et al., 2022).

Recently, machine learning has been widely utilized in the discriminative diagnosis using EEG signals in patients with MCI (Yang et al., 2019). (Lee et al. 2022) extracted spectral, nonlinear, and functional connectivity features from EEG signals in AD and MCI cases, achieving an accuracy of 86.85%. (Perez-Valero et al. 2022) applied spectral and nonlinear features from three groups—AD, MCI, and Healthy Controls (HC)—to an MLP classifier, achieving a maximum accuracy of 95%. Furthermore, researchers including (Li et al. 2022, 2021) and (Kim et al. 2022) focused their studies on the aMCI population. They employed machine learning models to classify aMCI against HC and non-aMCI groups, respectively, achieving an accuracy approaching 90%. Although researchers have attained satisfactory classification results through feature refinement and model optimization, the substantial overlap in EEG features between MCI patients and AD or HC underscores considerable room for further enhancement in classification performance (Ding et al., 2022; Ieracitano et al., 2020).

Longitudinal data, also known as panel data, involves repeated measurements for individuals. In the medical field, longitudinal studies facilitate the capture of individual trends with dynamic trajectories beyond the scope of cross-sectional studies, providing significant benefits in early diagnosis, risk prediction, treatment planning, and prognosis assessment (Cascarano et al., 2023). Though several researchers have collected EEG signals in MCI patients and conducted longitudinal studies (Miraglia et al., 2020; Vecchio et al., 2018), there is a lack of research extracting features from multiple EEG measurements and constructing longitudinal features. In the “HC-MCI-AD” disease continuum, the progression of the condition represents a dynamic evolving process (Monllor et al., 2021). Extracting longitudinal features can offer multidimensional longitudinal information for early diagnosis and prognosis improvement.

This study utilizes a prospective cohort design, recruiting aMCI participants and conducting a one-year follow-up. After the follow-up period, patients were categorized into SMCI and PMCI groups based on whether they progressed to AD, which was in alignment with definitions from previous research (Rossini et al., 2006; Vecchio et al., 2018). Each patient underwent a total of four EEG signal measurements at equidistant intervals, with spectral, nonlinear, and functional connectivity features extracted at each time point. Subsequently, the longitudinal features were constructed to capture the dynamic trends between the first three cross-sectional assessments. Lastly, extracted longitudinal features were integrated into various machine learning classifiers to predict one-year follow-up outcomes. Our study aimed to explore the advantages of longitudinal EEG features, furthermore demonstrating the necessity of repeated EEG measurements in longitudinal studies involving the AD disease continuum.

## 2 Methods

**Figure 1:**
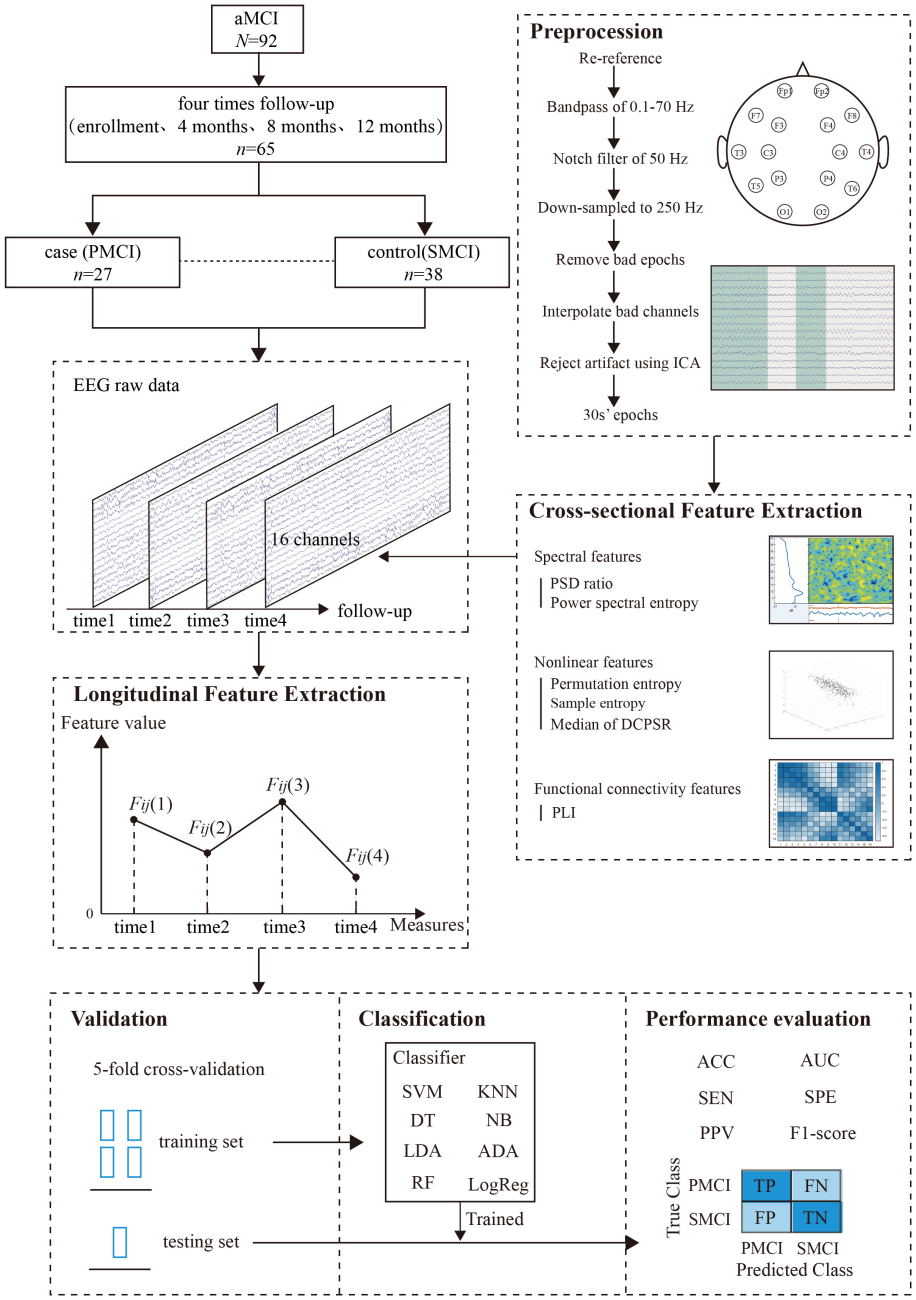
Study design.

The study design of our prediction framework is shown in Figure 1, which consists of five main steps: EEG data acquisition, EEG preprocessing, feature extraction, prediction, and evaluation.

### 2.1 Cohort

#### 2.1.1 General study design

Between July 1, 2022, and September 22, 2024, conducting a prospective cohort design, we recruited 92 aMCI patients from the Neurology Department of the First Affiliated Hospital of Sun Yat-sen University (SYSU). Within this cohort, 65 patients underwent EEG recordings every four months during the one-year follow-up, resulting in complete sets of four EEG records. As of the follow-up endpoint on September 22, 2024, 27 aMCI patients progressed to AD (PMCI), while 38 aMCI patients did not (SMCI). A total of 65 patients (27 PMCI, 38 SMCI) were included for our analysis.

#### 2.1.2 Definitions of SMCI and PMCI

We recruited aMCI cases and employed a one-year follow-up. Based on whether they progressed to AD after one year, they were categorized into the PMCI and SMCI groups. The diagnosis of aMCI was based on the Petersen 2011 criteria (Petersen, 2004). The inclusion criteria for this study were as follows: (1) age of 50 years and above, (2) memory complaint usually corroborated by an informant, (3) objective memory impairment for age, (4) essentially preserved general cognitive function, (5) largely intact functional activities. The exclusion criteria were: other forms of dementia or accompanying Parkinson's disease, epilepsy, psychiatric disorders, and serious organic disease. The diagnosis of AD was based on the criteria provided by the National Institute on Aging and the Alzheimer's Association (NIA-AA) in 2011 (McKhann et al., 2011). All diagnoses of aMCI and AD were established by experienced neurologists in strict accordance with the corresponding diagnostic criteria. The demographic information of the patients is shown in Table 1.

**Table 1:** The demographic characteristics of participants.

**Variable**	**SMCI (*n* = 38)**	**PMCI (*n* = 27)**	**Statistics**
Age (years)	68.97 ± 7.71	69.63 ± 7.65	*t* = −0.49, *P* = 0.62
Gender (male/female)	14/24	11/16	χ^2^= 0.10, *P* = 0.75
MMSE (*t*_1_, scores)	23.32 ± 1.83	23.70 ± 1.66	*t* = 0.87, *P* = 0.39
MoCA (*t*_1_, scores)	19.82 ± 2.23	19.44 ± 1.87	*t* = 0.71, *P* = 0.48
MMSE (*t*_1_−*t*_4_, scores)	1.32 ± 1.77	3.96 ± 1.37	*t* = 6.50, *P* < 0.001
MoCA (*t*_1_−*t*_4_, scores)	1.50 ± 1.54	5.30 ± 0.87	*t* = 11.57, *P* < 0.001

MMSE, mini-mental state examination; MoCA, montreal cognitive assessment; PMCI, progressive mild cognitive impairment; SMCI, stable mild cognitive impairment.

### 2.2 EEG data acquisition

The patients had the collection of resting-state EEG signals at four-month intervals, with a margin of ±5 days. Resting-state EEG was recorded using the Nicolet EEG machine system (Natus Medical Inc., San Carlos, CA) with a sampling rate of 500 Hz. Electrodes were positioned according to the 10–20 international system, with a total of 16 channels (Fp1, Fp2, F3, F4, C3, C4, P3, P4, O1, O2, F7, F8, T3, T4, T5, and T6). All patients were right-handed, and sufficient sleep was ensured the night before the EEG collection. During recording, patients were instructed to maintain a comfortable seated posture with their eyes closed for 5 minutes. EEG technicians continuously monitored the EEG traces and promptly alerted participants if any signs of drowsiness were detected.

### 2.3 EEG preprocessing

EEG signals are susceptible to various artifacts, highlighting the importance of preprocessing prior to analysis. Firstly, the raw EEG data were re-referenced using an average reference, and a finite impulse response (FIR) band-pass filter was applied to filter the EEG signals within the range of 0.1–70 Hz. Also, a notch filter was used to eliminate the 50 Hz power line interference. The EEG signals were subsequently down-sampled to 250 Hz. After joint screening by two experienced EEG examiners, bad epochs were removed and bad channels were interpolated. Then, 30-second segments of continuous EEG signals with clear background rhythms and minimal interference were selected. Following, we conducted independent component analysis (ICA) to remove common artifacts such as blinks, eye movements, and cardiac interference. Examiners applied the same procedure and criteria for preprocessing each EEG signal. The above preprocessing steps were all performed using the EEGLAB toolbox (Delorme and Makeig, 2004) in MATLAB (R2023a, MathWorks).

### 2.4 Feature extraction

Firstly, we extracted various cross-sectional features from each EEG signal, with each feature measured three times within the initial nine months, constituting repeated measures data. Subsequently, longitudinal features were extracted from the cross-sectional features.

#### 2.4.1 Cross-sectional feature

For each 30s EEG signal, we extracted features in three feature sets: spectral, nonlinear, and functional connectivity.

##### 2.4.1.1 Spectral feature

Using Welch's power spectral density (PSD) estimation (Alam et al., 2020), we transformed the preprocessed EEG signals from the time domain into the frequency domain and divided them into the following five subbands: delta (0.5–4 Hz), theta (4–8 Hz), alpha (8–13 Hz), beta (13–30 Hz), and gamma (30–45 Hz).

(1) Power spectral density ratio (PSD ratio): Considering the variation in absolute PSD values among different patients, we calculated the relative PSD values within the aforementioned subbands for each patient, resulting in the following five ratios:


$$\begin{array}{l} Ratio1=\frac{delta}{alpha} \end{array}$$ (1)

$$\begin{array}{l} Ratio2=\frac{theta}{alpha} \end{array}$$ (2)

$$\begin{array}{l} Ratio3=\frac{delta}{alpha+beta} \end{array}$$ (3)

$$\begin{array}{l} Ratio4=\frac{theta}{alpha+beta} \end{array}$$ (4)

$$\begin{array}{l} Ratio5=\frac{delta+theta}{alpha+beta+gamma} \end{array}$$ (5)

(2) Power spectral density entropy (PSDE): In each subband, a sequence of PSD values can be obtained. We used the Shannon entropy method to assess the level of disorder in this sequence of PSD values (Li et al., 2023). Assuming there are PSD series with *N* values within the subband, the PSDE was calculated as follows:


$$\begin{array}{l} E=-\overset{N}{\underset{i=1}{\sum }}p_{i}logp_{i} \end{array}$$ (6)

where *E* and *p*_*i*_ represent the PSDE of the signal and the probability of having the *i* sample in the signal, respectively.

##### 2.4.1.2 Nonlinear feature

We extracted the following three nonlinear features to capture the nonlinear characteristics of the EEG signals in aMCI patients.

(1) Permutation entropy (PE): PE is an efficient quantitative complexity measure that explores the local order structure of a dynamic time series (Bandt and Pompe, 2002), particularly in EEG signals from MCI and AD patients (Seker et al., 2021; Siuly et al., 2020). Given a scalar time series, {*x*(*i*):1 ≤ *i* ≤ *N*}. Firstly, reconstruct time series:


$$\begin{array}{l} X_{i}=\left\{x\left(i\right),x\left(i+\tau \right),\ldots ,x\left(i+\left(m-1\right)\tau \right)\right\} \end{array}$$ (7)

$$\begin{array}{l} \;i=1,\;2,\;\ldots ,N-\left(m-1\right)\tau \end{array}$$

where τ is time delay, and *m* is the embedding dimension.

Then, rearrange *X*_*i*_ in an increasing order:


$$\begin{array}{l} \{x\left(i+\left(j_{1}-1\right)\tau \right)\leq x\left(i+\left(j_{2}-1\right)\tau \right)\leq \ldots \leq \\ x\left(i+\left(j_{m}-1\right)\tau \right)\} \end{array}$$ (8)

There are *m*! permutations for *m* dimensions. Each vector *X*_*i*_ can be mapped to one of the *m*! permutations.

Next, the probability of the *j*_*th*_ permutation occurring *P*_*j*_ can be defined as:


$$\begin{array}{l} P_{j}=\frac{n_{j}}{\sum_{j=1}^{m!}n_{j}} \end{array}$$ (9)

where *n*_*j*_ is the number of times the *j*_*th*_ permutation is occurring.

The permutation entropy of the time series {*x*(*i*):1 ≤ *i* ≤ *N*} is defined by:


$$\begin{array}{l} H_{x}\left(m\right)=-\overset{m!}{\underset{j=1}{\sum }}P_{j}lnP_{j} \end{array}$$ (10)

when the time series is random, the *H*_*x*_(*m*) approaches its maximum value of ln(*m*!); when the time series is regular, the *H*_*x*_(*m*) approaches zero.

Finally, normalizing *H*_*x*_(*m*) by dividing ln(*m*!):


$$\begin{array}{l} PE=\frac{H_{x}\left(m\right)}{\ln\left(m!\right)} \end{array}$$ (11)

(2) Sample entropy (SE): The SE is proposed by Richman and Moorman (Richman and Moorman, 2000) as an improvement over approximate entropy (AE), addressing the bias present in AE. Recently, SE has been extensively utilized for feature extraction in patients with MCI and AD (Ruiz-Gómez et al., 2018; Tsai et al., 2012).

Assume we have a time-series data set of length *N* = {*x*_1_, *x*_2_, …, *x*_*N*_} with a constant time interval τ. We define a template vector of length *m*, such that *X*_*M*_(*i*) = {*x*_*i*_, *x*_*i*+1_, *x*_*i*+2_, …, *x*_*i*+*m*−1_} and the distance function ||*X*_*m*_(*i*), *x*_*m*_(*j*)|| (*i*≠*j*) is to be the Chebyshev distance. We define the SE to be


$$\begin{array}{l} SE=-\ln\frac{A}{B} \end{array}$$ (12)

where *A* = number of template vector pairs having ||*X*_*m*+1_(*i*), *x*_*m*+1_(*j*)|| < *r*, and *B* = number of template vector pairs having ||*X*_*m*_(*i*), *x*_*m*_(*j*)|| < *r*. We take the value of *m* to be 2 and the value of *r* to be 0.2 × Std.

(3) Median distance from the centroid of phase space reconstruction (M-DCPSR): Phase space reconstruction (PSR) is applied in EEG research to unveil the nonlinear dynamical properties and spatiotemporal relationships of brain electrical activity (Kaur et al., 2020; Lee et al., 2014). We innovatively propose M-DCPSR to unveil the nonlinear characteristics of EEG in the aMCI population.

Assume we have a time-series data set of length *N* = {*x*_1_, *x*_2_, …, *x*_*N*_}, the calculation is as follows:

(3.1) Setting the embedding dimension of PSR as *m* = 3 and determine the lag of the time series (τ) using the autocorrelation function:


$$\begin{array}{l} N=N-x_{mean}=\{x_{1}-x_{mean},x_{2-x_{mean}},\ldots ,\\ \;x_{N}-x_{mean}\} \end{array}$$ (13)

$$\begin{array}{l} SSd=N\cdot N \end{array}$$ (14)

$$\begin{array}{l} R_{xx}\left(\tau \right)=\frac{1}{SSd}\overset{N-\tau }{\underset{i=1}{\sum }}N\left(i\right)\cdot N\left(i+\tau \right) \end{array}$$ (15)

By detecting zero crossings of the autocorrelation function, the time delay τ is identified. The values of the autocorrelation function are iterated through in a loop, and when


$$\begin{array}{l} R_{xx}\left(j-1\right)\cdot R_{xx}\left(j\right)\leq 0 \end{array}$$ (16)

it indicates a zero crossing in the autocorrelation function. Subsequently, the time delay τ is determined based on the absolute values of the autocorrelation function before and after the crossing point:


$$\{\begin{array}{ll} \tau =j, & \left|R_{xx}(j-1)\right|\leq \left|R_{xx}(j)\right|\\ \tau =j-1, & \left|R_{xx}(j-1)\right|>\left|R_{xx}(j)\right| \end{array}$$ (17)

(3.2) Construct the three-dimensional coordinates of the time series in the phase space based on the τ:


$$\begin{array}{c} \left(x_{n},y_{n},z_{n}\right)=\left\{\begin{array}{ccc} x_{1} & x_{1+\tau } & x_{1\;+\;2\tau }\\ x_{2} & x_{2+\tau } & x_{2\;+\;2\tau }\\ \begin{array}{c} \vdots \\ x_{N-2\tau } \end{array} & \begin{array}{c} \vdots \\ x_{N-\tau } \end{array} & \begin{array}{c} \vdots \\ x_{N} \end{array} \end{array}\right\} \end{array}$$ (18)

each column of matrix represents one dimension of a three-dimensional coordinate.

(3.3) Calculate the centroid of the structure formed by all points in the phase space:


$$\begin{array}{l} \begin{array}{c} \left(\frac{\sum_{i=1}^{n}x_{i}}{n},\frac{\sum_{i=1}^{n}y_{i}}{n},\frac{\sum_{i=1}^{n}z_{i}}{n}\right) \end{array} \end{array}$$ (19)

(3.4) Calculate the Euclidean distance between each point and the centroid:


$$\begin{array}{l} \begin{array}{c} d_{n}=\sqrt{{\left(x_{n}-\frac{\sum_{i=1}^{n}x_{i}}{n}\right)}^{2}+{\left(y_{n}-\frac{\sum_{i=1}^{n}y_{i}}{n}\right)}^{2}+{\left(z_{n}-\frac{\sum_{i=1}^{n}z_{i}}{n}\right)}^{2}} \end{array} \end{array}$$ (20)

(3.5) Finally, calculate the median of these Euclidean distances, yielding the M-DCPSR for the given time series.

##### 2.4.1.3 Functional connectivity feature

Phase lag index (PLI), which is used to measure the degree of phase synchronization between two signals, can exclude the influences of volume conduction in EEG signals. It is commonly employed as a functional connectivity feature in MCI and AD patients (Kuang et al., 2022; Nobukawa et al., 2020; Núñez et al., 2019). PLI values range from 0 to 1. A PLI of zero indicates either no coupling or coupling with a phase difference centered around 0 or π. A PLI of 1 indicates perfect phase locking at a value different from 0 or π. Due to the significant alterations observed in the alpha band among the MCI population, this study exclusively extracted the PLI within the alpha band from the patients.

Suppose the band-pass filtered signals at two electrodes are *X*(*t*) and *Y*(*t*), respectively. Through the Hilbert transform, we can obtain their analytic signals *X*_*an*_(*t*) and *Y*_*an*_(*t*), as


$$\begin{array}{l} X_{an}\left(t\right)=X\left(t\right)+iX_{H}\left(t\right) \end{array}$$ (21)

$$\begin{array}{l} Y_{an}\left(t\right)=Y\left(t\right)+iY_{H}\left(t\right) \end{array}$$ (22)

where *X*_*H*_(*t*) and *Y*_*H*_(*t*) are the Hilbert transform of band-pass filtered signals *X*(*t*) and *Y*(*t*), respectively. Namely,


$$\begin{array}{l} X_{H}\left(t\right)=\frac{1}{\pi }P.V.\int_{-\infty }^{+\infty }\frac{X\left(\tau \right)}{t-\tau }d\tau \end{array}$$ (23)

$$\begin{array}{l} Y_{H}\left(t\right)=\frac{1}{\pi }P.V.\int_{-\infty }^{+\infty }\frac{Y\left(\tau \right)}{t-\tau }d\tau \end{array}$$ (24)

where *P. V*. is the Cauchy principal value.

Using analytical signals, the instantaneous amplitude *A*_*x*_(*t*), *A*_*y*_(*t*) and instantaneous phase ϕ_*x*_(*t*), ϕ_*y*_(*t*), can be calculated:


$$\begin{array}{l} A_{x}\left(t\right)=\sqrt{{X_{an}\left(t\right)}^{2}+{X_{H}\left(t\right)}^{2}} \end{array}$$ (25)

$$\begin{array}{l} A_{y}\left(t\right)=\sqrt{{Y_{an}\left(t\right)}^{2}+{Y_{H}\left(t\right)}^{2}} \end{array}$$ (26)

$$\begin{array}{l} \phi_{x}\left(t\right)=\tan^{-1}\frac{X_{H}\left(t\right)}{X_{an}\left(t\right)} \end{array}$$ (27)

$$\begin{array}{l} \phi_{y}\left(t\right)=\tan^{-1}\frac{Y_{H}\left(t\right)}{Y_{an}\left(t\right)} \end{array}$$ (28)

We calculate Δϕ_*xy*_(*t*), which is their phase difference at time *t*:


$$\begin{array}{l} \Delta \phi_{xy}\left(t\right)=|\phi_{x}\left(t\right)-\phi_{y}\left(t\right)| \end{array}$$ (29)

In actual analysis, the phase difference needs to be converted to [0, 2π):


$$\begin{array}{l} \Delta \phi_{rel}\left(t\right)=\Delta \phi_{xy}\left(t\right)mod2\pi \end{array}$$ (30)

Finally, the formula of PLI is:


$$\begin{array}{l} PLI=\left|\frac{1}{N}\overset{N}{\underset{N=1}{\sum }}sign\left(\Delta \phi_{rel}\left(t\right)\right)\right| \end{array}$$ (31)

#### 2.4.2 Longitudinal feature

We extracted features related to the centralized, dispersion, and dynamic trends of each EEG cross-sectional feature across the initial three measurements, constituting the longitudinal features.

For the *t*_*th*_ time point, a panel dataset *X*_*ij*_(*t*) is formed for the *j*_*th*_ independent variable collected from patient *i*. Assuming there are *N* patients, each with records for *P* variables across *T* periods.

##### 2.4.2.1 Centralized trend

The centralized feature is denoted as:


$$\begin{array}{l} MF\left(F_{ij}\right)=\frac{\sum_{i=1}^{T}X_{ij}\left(t\right)}{T} \end{array}$$ (32)

*MF*(*F*_*ij*_) refers to the mean value of the *j*_*th*_ independent variable collected from patient *i* over the entire period *T*. This feature reflects the average level of the *j*_*th*_ independent variable collected for individual *i* throughout the entire period.

##### 2.4.2.2 Dispersion trend

The dispersion features are denoted as:


$$\begin{array}{l} SDF\left(F_{ij}\right)={\left[\frac{\sum_{t=1}^{T}{\left(X_{ij}\left(t\right)-{\bar{X}}_{ij}\right)}^{2}}{T-1}\right]}^{\frac{1}{2}} \end{array}$$ (33)

$$RF\left(F_{ij}\right)=\left(F_{ij}\right)-\left(F_{ij}\right)$$ (34)

where ${\bar{X}}_{ij}$ represents the *MF*(*F*_*ij*_), *SDF*(*F*_*ij*_) and *RF*(*F*_*ij*_) reflect the dispersion level of the *j*_*th*_ independent variable collected for individual *i* throughout the entire period.

##### 2.4.2.3 Dynamic trend

The combination of numerical and graphical features reflecting the dynamic trend are denoted as:


$$\begin{array}{l} AF\left(F_{ij}\right)=AREA(F_{ij}\left(1\right)-F_{ij}\left(2\right)-F_{ij}\left(3\right)-time1\\ \;-time3) \end{array}$$ (35)

$$\begin{array}{l} Velocity\left(F_{ij}\right)=\frac{X_{ij}\left(t+1\right)-X_{ij}\left(t\right)}{\delta t} \end{array}$$ (36)

$$\begin{array}{l} MVF\left(F_{ij}\right)=\frac{\sum_{i=1}^{T}Velocity_{ij}\left(t\right)}{T} \end{array}$$ (37)

$$\begin{array}{l} SDVF\left(F_{ij}\right)={\left[\frac{\sum_{t=1}^{T}{\left(Velocity_{ij}\left(t\right)-{\bar{Velocity}}_{ij}\right)}^{2}}{T-1}\right]}^{\frac{1}{2}} \end{array}$$ (38)

where *AF*(*F*_*ij*_) is the area enclosed by the five points *F*_*ij*_(1), *F*_*ij*_(2), *F*_*ij*_(3), time1, and time3 in Figure 1. This area is the sum of the three trapezoidal areas formed by the feature values at adjacent time points. The horizontal axis represents the collection time (months), and the vertical axis represents the magnitude of the original feature values. *Velocity*(*F*_*ij*_) refers to the rate of change between two consecutive measurements of the *j*_*th*_ independent variable for patient *i* at time points *t* and *t* – 1. It corresponds to the slopes of the lines connecting *F*_*ij*_(1) and *F*_*ij*_(2), *F*_*ij*_(2) and *F*_*ij*_(3) in Figure 1. *MVF*(*F*_*ij*_) represents the mean of these three slope values, and *SDVF*(*F*_*ij*_) represents their standard deviation.

As shown in Table 2, for each cross-sectional feature, we extracted six longitudinal features, resulting in a total of 1968 longitudinal features. The aforementioned extracted longitudinal features were fed into the machine learning classifiers.

**Table 2:** Number of extracted features.

**Longitudinal features**	**Feature sets**	**Features**	**Number of features**
^*^ MF, SDF, RF, AF, MVF, SDVF	Spectral	PSD ratio	5 types × 16 channels = 80
		PSD entropy	5 bands × 16 channels = 80
	Complexity	Permutation entropy	16 channels
		Sample entropy	16 channels
		Median of DCPSR	16 channels
	Connectivity	Phase lag index	162=120 pairs

MF, mean; SDF, standard deviation; RF, range; AF, area under the feature curve; MVF, the mean of the velocity; SDVF, the standard deviation of the velocity; PSD, power spectral density; DCPSR, distance from the centroid of phase space reconstruction.

#### 2.4.3 Feature selection

To mitigate the risk of overfitting resulting from the high-dimensional feature space, a filter feature selection method was used. During each training phase, the *F*-statistic was computed for every feature using only the training data. Features were ranked by descending *F*-value, and the top 100 were retained for classifier development.

### 2.5 Statistical analysis

For comparisons of cross-sectional features between the SMCI and PMCI groups at a single time point, the Mann-Whitney *U* test was applied, as some features did not meet the assumptions required for parametric testing. To further examine whether the trajectories of these features over time differed between the two groups, linear mixed models (LMM) were applied.

In the LMM, group (SMCI vs. PMCI), time (time1, time2, time3, and time4), and their interaction (group × time) were included as fixed effects, while random intercepts for each subject were introduced to account for within-subject correlations due to repeated measurements. This approach enabled direct comparison of longitudinal feature trajectories between groups while addressing the non-independence of repeated measurements. All analyses were conducted using MATLAB R2023a and R 4.3.2, with a significance level of α = 0.05.

### 2.6 Prediction and validation

We employed eight commonly used machine learning classifiers for one-year outcomes prediction between PMCI and SMCI, including support vector machine (SVM), decision tree (DT), naive bayes (NB), linear discriminant analysis (LDA), AdaBoost (ADA), *k*-nearest neighbor (KNN), random forest (RF), and logistic regression (LogReg). All the parameters for machine learning models were set to the default settings in MATLAB. To evaluate model performance robustly, a repeated 10 × 5-fold cross-validation (CV) scheme was employed. Finally, we assessed the classification performance of the machine learning model using six metrics: SEN, SPE, PPV, F1-score, ACC, and AUC. The formula for the previously mentioned metrics is as follows:


$$\begin{array}{l} ACC=\frac{TP+TN}{TP+FN+TN+FP} \end{array}$$ (39)

$$\begin{array}{l} SEN=\frac{TP}{TP+FN} \end{array}$$ (40)

$$\begin{array}{l} SPE=\frac{TN}{FP+TN} \end{array}$$ (41)

$$\begin{array}{l} PPV=\frac{TP}{TP+FP} \end{array}$$ (42)

$$\begin{array}{l} F1-score=\frac{2TP}{2TP+FP+FN} \end{array}$$ (43)

$$\begin{array}{l} AUC=\frac{\sum_{ins_{i}\in positiveclass}rank_{ins_{i}}-\frac{M\times \left(M+1\right)}{2}}{M\times N} \end{array}$$ (44)

where *M, N* are the number of positive sample and negative sample, separately. *TP* is the number of PMCI cases that are correctly predicted, *FN* is the number of PMCI cases that are incorrectly predicted as SMCI samples, *FP* is the number of SMCI cases that are incorrectly predicted as PMC cases, and *TN* is the number of SMCI samples that are correctly predicted.

## 3 Results

### 3.1 Spectral feature

**Figure 2:**
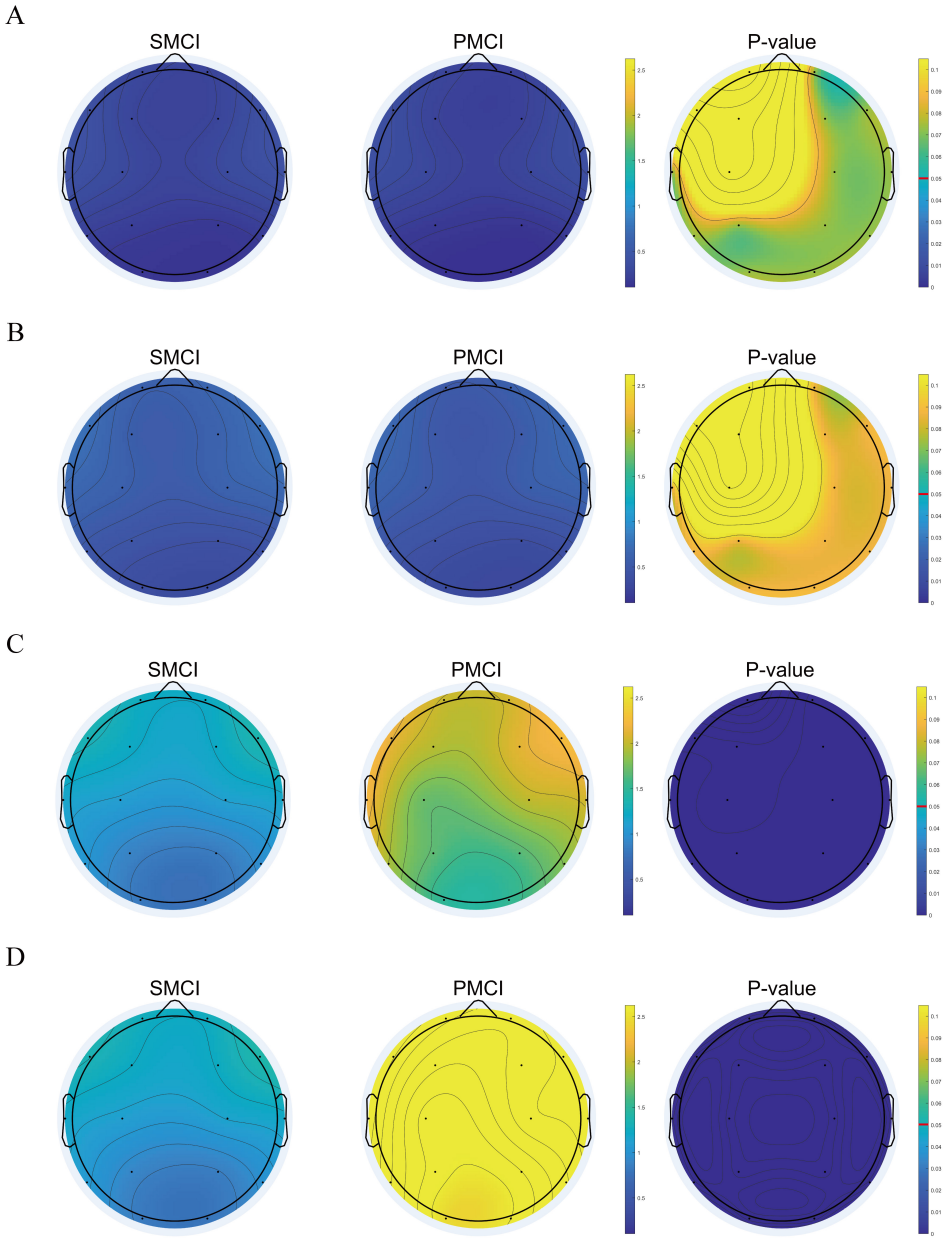
The topoplot of original value and statistical differences for Power spectral density (PSD) Ratio2 in stable mild cognitive impairment (SMCI) and progressive mild cognitive impairment (PMCI) groups including 0 month **(A)**, 4 months **(B)**, 8 months **(C)**, and 12 months **(D)**. The first and second column of each sub-figure are the topoplots of mean in SMCI and PMCI groups. The third column of each sub-figure is the topoplot of statistical differences in SMCI and PMCI groups.

As illustrated in Figure 2, at time1 and time2, there were no significant differences in PSD ratio2 between the PMCI and SMCI groups across the entire brain. However, at time3 and time4, the differences became statistically significant, with higher values observed in the PMCI group compared to the SMCI group. Similar results were obtained for PSD ratio1, ratio3, ratio4, and ratio5, as shown in Supplementary Appendix A.

At time1 and time2, there were no significant differences in the PSDE for delta, theta, alpha, beta, and gamma bands between the PMCI and SMCI groups across the entire brain. However, at time3 and time4, these differences were statistically significant. Specifically, in the delta and theta bands, PMCI exhibited higher PSDE compared to SMCI across the entire brain. In the alpha and beta bands, PMCI showed lower PSDE compared to SMCI across the entire brain. For the gamma band, PMCI showed higher PSDE in the central and parietal regions compared to SMCI, while in all other channels, it was lower than SMCI. The detailed results of PSDE were presented in Supplementary Appendix A.

### 3.2 Nonlinear feature

**Figure 3:**
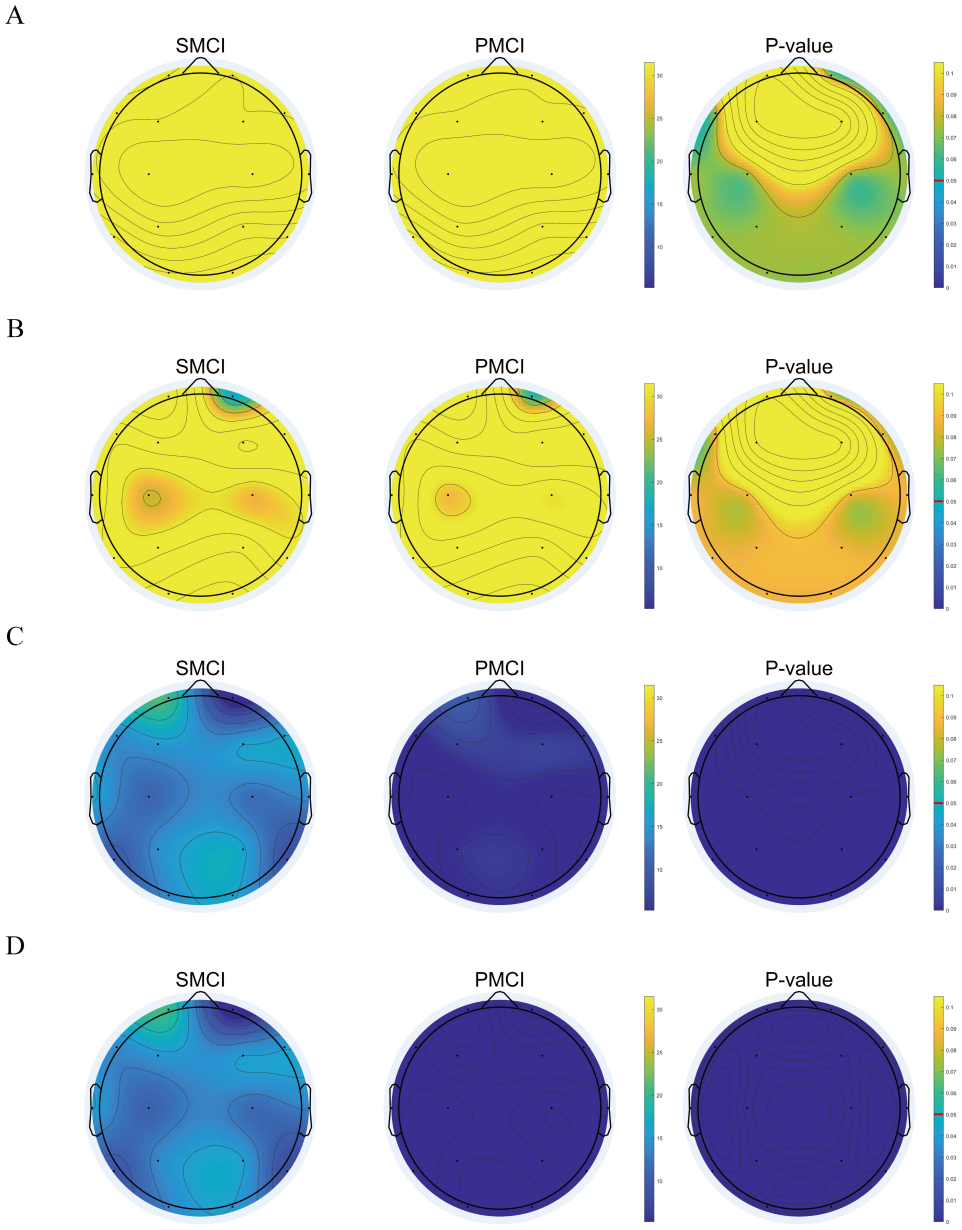
The topoplot of original value and statistical differences for Median distance from the centroid of phase space reconstruction (M-DCPSR) in stable mild cognitive impairment (SMCI) and progressive mild cognitive impairment (PMCI) groups including 0 month **(A)**, 4 months **(B)**, 8 months **(C)**, and 12 months **(D)**. The first and second column of each sub-figure are the topoplots of mean in SMCI and PMCI groups. The third column of each sub-figure is the topoplot of statistical differences in SMCI and PMCI groups.

As shown in Figure 3, at time1 and time2, there were no significant differences in the M-DCPSR between the PMCI and SMCI groups across the entire brain. However, at time3 and time4, these differences were statistically significant, with PMCI exhibiting lower values than SMCI. The results for SE and PE were consistent with those of M-DCPSR, as detailed in Supplementary Appendix A.

### 3.3 Functional connectivity feature

**Figure 4:**
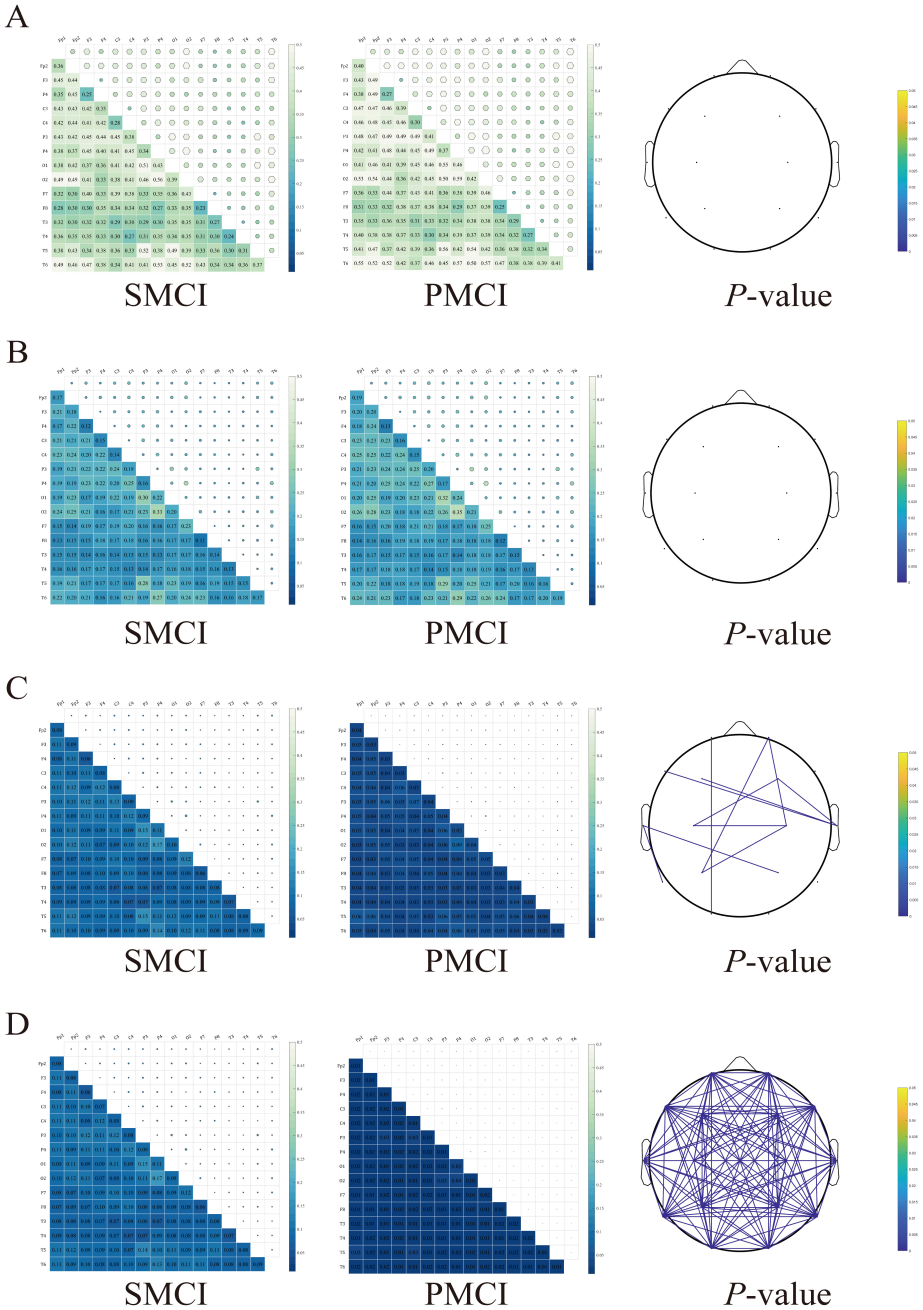
The combination chart of original value and statistical differences for Phase lag index (PLI) in stable mild cognitive impairment (SMCI) and progressive mild cognitive impairment (PMCI) groups including 0 month **(A)**, 4 months **(B)**, 8 months **(C)**, and 12 months **(D)**. The first and second column of each sub-figure are the heatmaps of mean in SMCI and PMCI groups. The third column of each sub-figure is the topoplot of connections with statistical differences in SMCI and PMCI groups.

As illustrated in Figure 4, at time1 and time2, there were no significant differences in the PLI between the PMCI and SMCI groups across the entire brain. However, at time3, the differences in connectivity between electrode pairs across the few brain regions were statistically significant. Furthermore, at time4, the differences in connectivity between most electrode pairs across the entire brain were statistically significant, with PMCI exhibiting lower values than SMCI.

### 3.4 Composition of selected features

The selection frequency of each feature type was aggregated across all iterations of the repeated 10 × 5-fold CV. The distribution was as follows: SDF (1,197, 23.94%), SDVF (1,033, 20.66%), RF (936, 18.72%), AF (923, 18.46%), and MVF (911, 18.22%). MF features were not selected.

### 3.5 Prediction performance

**Figure 5:**
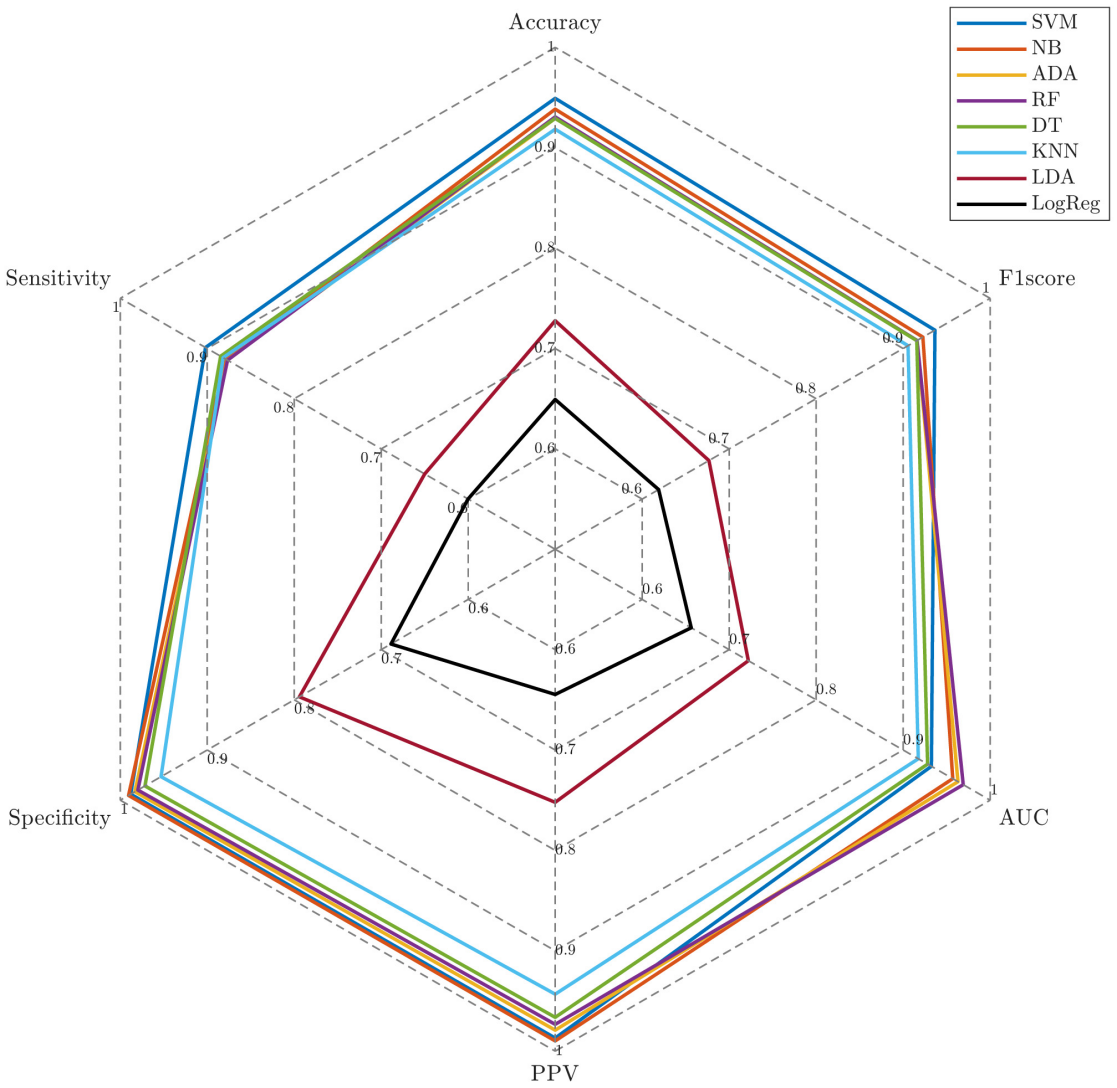
The radar chart of prediction results using repeated 10 × 5-fold cross-validation.

Figure 5 and Table 3 present the prediction performance of eight classifiers using the selected 100 longitudinal features under repeated 10 × 5-fold CV. SVM achieved the best overall performance, attaining the highest mean and lowest standard deviation across ACC (94.92%), SEN (90.20%), and F1-score (93.65%). NB exhibited the highest SPE (99.05%) and PPV (98.98%), while RF achieved the highest AUC (96.91%). ADA, DT and KNN performed remarkably well, with all metrics exceeding 80%. Despite LDA and LogReg having inferior performance, their evaluation metrics generally remained around 60–70%.

**Table 3:** The prediction results using repeated 10 × 5-fold CV (mean ± standard deviation, %).

**Classifier**	**ACC**	**AUC**	**SEN**	**SPE**	**PPV**	**F1-score**
SVM	**94.92** **±6.70**	93.25 ± 9.62	**90.20** **±13.56**	98.80 ± 4.15	98.70 ± 4.52	**93.65** **±9.11**
DT	92.92 ± 8.49	92.81 ± 8.82	88.46 ± 14.98	97.16 ± 7.61	96.63 ± 9.48	91.57 ± 11.13
NB	93.85 ± 7.77	95.71 ± 7.22	87.90 ± 15.87	**99.05** **±3.82**	**98.98** **±4.12**	92.27 ± 11.29
LDA	72.77 ± 16.02	72.20 ± 16.03	65.00 ± 23.23	79.41 ± 18.33	75.21 ± 19.76	67.67 ± 19.02
ADA	92.92 ± 8.63	96.30 ± 6.08	87.70 ± 15.94	98.31 ± 5.51	97.91 ± 6.55	91.60 ± 10.60
RF	93.08 ± 7.81	**96.91** **±6.02**	87.67 ± 14.77	97.98 ± 5.14	97.33 ± 7.10	91.60 ± 10.09
KNN	91.85 ± 8.43	91.74 ± 8.87	88.18 ± 14.33	95.31 ± 8.34	94.32 ± 9.86	90.56 ± 10.64
LogReg	64.92 ± 17.25	65.64 ± 18.02	60.02 ± 21.72	68.88 ± 21.37	64.47 ± 22.58	61.89 ± 17.74

Bold indicates the optimal value for predicted performance. CV, cross-validation; ACC, accuracy; AUC, area under the curve; SEN, sensitivity; SPE, specificity; PPV, positive predictive value; SVM, support vector machine; DT, decision tree; NB, naive bayes; LDA, linear discriminant analysis; ADA, AdaBoost; KNN, k-nearest neighbor; RF, random forest; LogReg, logistic regression.

### 3.6 Performance comparison

To spotlight the advantages of longitudinal features, we combined information from the initial three cross-sections to explore the necessity of repeated EEG measurements. The prediction performance for different cross-sectional combinations was presented in Supplementary Appendix B. The results demonstrated that the optimal classification performance was achieved when utilizing information from all the initial three cross-sections, followed by two cross-sections. This highlighted the enhanced model performance associated with constructing longitudinal features based on repeated EEG measurements.

## 4 Discussion

Conducting repeated EEG measurements during the follow-up period, this study comprehensively extracted cross-sectional features including spectral, nonlinear, and functional connectivity. By constructing longitudinal features that reflect dynamic trends and integrating machine learning methods, we established a prediction framework for both SMCI and PMCI populations, demonstrating robust predictive performance in internal validation.

The cross-sectional EEG features extracted in this study have been validated for their excellent discriminative ability across three groups: AD, MCI, and HC. We further focused our research on aMCI individuals, providing supporting evidence through follow-up studies. In this study, both groups exhibited an increasing trend in PSD ratios 1–5, as well as elevated PSDE values in the delta and theta bands. Conversely, a declining pattern was observed in PSDE values within the alpha and beta bands. These results substantiate a transition in EEG spectra from higher to lower frequencies in the MCI population, consistent with existing research conclusions (Ding et al., 2022; Kwak, 2006; Luckhaus et al., 2008; Prichep et al., 2006; Rossini et al., 2006; Yan et al., 2021). We innovatively proposed the non-linear metric M-DCPSR. Both groups exhibit varying degrees of decreasing trends in M-DCPSR, SE, and PE, confirming the reduced complexity of EEG signals in the MCI population from a follow-up study perspective (Deng et al., 2017; Mammone et al., 2018; Tsai et al., 2012; Wang et al., 2019). Additionally, both groups exhibit distinctive decreasing patterns in the PLI with the alpha band, indicating diverse progression in intra- and inter-regional disconnect phenomena, consistent with relevant research findings (Núñez et al., 2019; Ruiz-Gómez et al., 2019). The aforementioned three phenomena may be attributed to the loss of neurons, the altered anatomical structure of neuronal tracts, and the modified release of neurotransmitters (Delbeuck et al., 2003; Olsson et al., 2016; Uhlhaas and Singer, 2006).

An interesting finding of our research is that, at time1 and time2, there were no significant differences in most EEG features between the two groups. However, at time3 and time4, significant differences were observed in the majority of EEG features, indicating that the changing trends of the extracted EEG features in the two groups were inconsistent. This observation is consistent with the high-risk nature of our cohort, as all participants were recruited from the Neurology Department of the First Affiliated Hospital of Sun Yat-sen University, a regional referral center for neurological diseases in South China, where patients are predominantly referred from primary hospitals and represent a high-risk aMCI population with an increased likelihood of short-term progression. These time-dependent differences support the necessity of conducting repeated EEG measurements during follow-up and constructing longitudinal features.

(Mubeen et al. 2017) and (Ardekani et al. 2017) utilized two follow-up MRI measurements from MCI patients and constructed longitudinal features, including the rate of change. Their studies demonstrated an improvement in classification performance when employing longitudinal features compared to cross-sectional features. Our study effectively demonstrates the crucial role of longitudinal features in predicting the disease progression of patients for the AD continuum. Not only does this study extract statistical indicators such as mean, standard deviation, and range, reflecting central tendency and dispersion, but it also utilizes the concept of “combining numerical and graphical information” to extract longitudinal indicators such as the area under the feature curve and the mean and standard deviation of the velocity, further enhancing the classification performance. Feature selection revealed that metrics capturing variability (SDF and SDVF) and change magnitude (RF, AF, and MVF) predominated, whereas mean (MF) features were entirely excluded—suggesting that EEG signal instability and dynamic evolution are more informative than static averages for short-term progression prediction. Due to the gradual yet dynamic progression of MCI to AD, incorporating longitudinal features enhances the performance compared to relying solely on cross-sectional features, further emphasizing the significance of longitudinal studies (Miraglia et al., 2020; Vecchio et al., 2018). The differing trajectories of EEG features among the MCI population confirm the heterogeneity of EEG signals in this group. Our study design can be extended to other age-related diseases or neurological disorders, as well as EEG signal analysis, and various medical research domains.

Incorporating the selected longitudinal features into machine learning models, the SVM classifier demonstrated the best overall prediction performance. This aligns with its strength in modeling complex, nonlinear relationships even with limited samples (Brereton and Lloyd, 2010; Meng and Zhao, 2015), a characteristic pertinent to the dynamic nature of longitudinal EEG data. NB excelled specifically in specificity and positive predictive value, suggesting that certain EEG-derived features exhibit strong class-conditional independence beneficial for ruling out progression (Raschka, 2014). RF achieved the highest AUC, its robust performance is consistent with its known capability to handle high-dimensional data and mitigate overfitting through ensemble learning (Capitaine et al., 2021; Wang et al., 2018). In contrast, linear classifiers including LDA and LogReg showed the lowest discrimination, reflecting their inherent limitation in capturing nonlinear patterns in EEG data (Günther et al., 2012; Wagner et al., 2021; Zhang et al., 2013). The fact that all nonlinear classifiers (SVM, RF, NB, ADA, DT, and KNN) achieved metrics exceeding 80% underscores the general utility and discriminative value of the constructed longitudinal EEG features for prediction within the aMCI continuum.

From the perspective of early prediction, this study adopted a prospective cohort design, constructed EEG longitudinal features, and combined machine learning methods to establish a prediction framework for both SMCI and PMCI populations, achieving satisfactory performance. However, several limitations should be acknowledged. First, the sample size was relatively small, and all participants were recruited from a single tertiary center, which may affect the generalizability of our findings. Second, this study did not employ the Clinical Dementia Rating (CDR) scale, which is a well-established and widely used tool for staging functional impairment in the Alzheimer's disease continuum (Angioni et al., 2024). Third, the follow-up duration was one year, which is a relatively short interval within the prolonged course of AD. While a high conversion rate was observed in this enriched cohort, longer follow-up is needed to evaluate the stability of the predictive framework over time. Fourth, the performance metrics reported here are derived from internal repeated cross-validation. The absence of an independent external test set may lead to optimistic estimates of real-world generalizability (Kikuchi et al., 2022).

To address these limitations, we are expanding the cohort through a multi-center collaboration, establishing additional cohorts at Sun Yat-sen Memorial Hospital and the Third Affiliated Hospital of Sun Yat-sen University to form an independent validation dataset. We also plan to extend the follow-up period to further evaluate the long-term predictive stability of the framework. Future work will additionally focus on enhancing interpretability and the integration of multimodal biomarkers to enhance clinical applicability.

## 5 Conclusion

Aiming to facilitate the dynamic tracking of disease progression in aMCI patients, we introduced a prediction framework utilizing EEG longitudinal features and machine learning methods in SMCI and PMCI populations. The framework not only comprehensively extracted spectral, nonlinear, and functional connectivity features from EEG data, but also further constructed longitudinal features reflecting dynamic trends. The SVM classifier achieved an accuracy of 94.92%, with most classifiers demonstrating satisfactory classification performance, indicating the stability of this framework. Our study captured the dynamic changes in EEG features among the aMCI population, providing robust follow-up evidence for the trajectory of aMCI individuals and offering EEG biomarkers with predictive value for AD.

## Data Availability

The raw data supporting the conclusions of this article will be made available by the authors, without undue reservation.
