# Supplementary material for: Predicting progression from amnestic mild cognitive impairment to Alzheimer's disease using longitudinal EEG data: a 12-month cohort study
Source: Front Aging Neurosci. 2026 Jan 20;17:1719981. doi: 10.3389/fnagi.2025.1719981 (PMC12864486; doi:10.3389/fnagi.2025.1719981)
Supplement: Supplementary file 1 [file Table_1.docx]

| **Supplementary Table 1** The results of the interaction effect test in the  linear mixed model for the PSD Ratio1 feature set | | | |
| --- | --- | --- | --- |
| channel | $\hat{\beta}$ | *t* | *P* |
| Fp1 | 1.426 | 14.157 | <0.001 |
| Fp2 | 1.589 | 14.857 | <0.001 |
| F3 | 1.233 | 14.620 | <0.001 |
| F4 | 1.119 | 14.571 | <0.001 |
| C3 | 1.032 | 15.375 | <0.001 |
| C4 | 1.121 | 15.636 | <0.001 |
| P3 | 1.076 | 15.418 | <0.001 |
| P4 | 1.086 | 14.689 | <0.001 |
| O1 | 0.891 | 15.653 | <0.001 |
| O2 | 1.000 | 14.780 | <0.001 |
| F7 | 1.357 | 15.967 | <0.001 |
| F8 | 1.810 | 15.818 | <0.001 |
| T3 | 1.495 | 17.447 | <0.001 |
| T4 | 1.412 | 16.667 | <0.001 |
| T5 | 1.083 | 16.410 | <0.001 |
| T6 | 1.839 | 15.805 | <0.001 |

PSD: Power spectral density

| **Supplementary Table 2** The results of the interaction effect test in the  linear mixed model for the PSD Ratio2 feature set | | | |
| --- | --- | --- | --- |
| channel | $\hat{\beta}$ | *t* | *P* |
| Fp1 | 0.624 | 15.281 | <0.001 |
| Fp2 | 0.603 | 17.422 | <0.001 |
| F3 | 0.539 | 14.811 | <0.001 |
| F4 | 0.613 | 17.152 | <0.001 |
| C3 | 0.492 | 15.651 | <0.001 |
| C4 | 0.649 | 17.257 | <0.001 |
| P3 | 0.563 | 17.300 | <0.001 |
| P4 | 0.594 | 17.201 | <0.001 |
| O1 | 0.515 | 16.349 | <0.001 |
| O2 | 0.562 | 15.541 | <0.001 |
| F7 | 0.655 | 16.408 | <0.001 |
| F8 | 0.600 | 16.747 | <0.001 |
| T3 | 0.738 | 17.784 | <0.001 |
| T4 | 0.674 | 17.501 | <0.001 |
| T5 | 0.637 | 16.547 | <0.001 |
| T6 | 0.644 | 16.627 | <0.001 |

PSD: Power spectral density

| **Supplementary Table 3** The results of the interaction effect test in the  linear mixed model for the PSD Ratio3 feature set. | | | |
| --- | --- | --- | --- |
| channel | $\hat{\beta}$ | *t* | *P* |
| Fp1 | 0.993 | 14.082 | <0.001 |
| Fp2 | 1.074 | 14.699 | <0.001 |
| F3 | 0.889 | 15.283 | <0.001 |
| F4 | 0.765 | 15.161 | <0.001 |
| C3 | 0.772 | 15.860 | <0.001 |
| C4 | 0.795 | 15.839 | <0.001 |
| P3 | 0.777 | 14.996 | <0.001 |
| P4 | 0.824 | 14.438 | <0.001 |
| O1 | 0.616 | 15.586 | <0.001 |
| O2 | 0.662 | 15.783 | <0.001 |
| F7 | 0.949 | 15.740 | <0.001 |
| F8 | 1.209 | 15.408 | <0.001 |
| T3 | 1.080 | 17.615 | <0.001 |
| T4 | 0.962 | 17.516 | <0.001 |
| T5 | 0.758 | 16.676 | <0.001 |
| T6 | 1.227 | 16.168 | <0.001 |

PSD: Power spectral density

| **Supplementary Table 4** The results of the interaction effect test in the  linear mixed model for the PSD Ratio4 feature set | | | |
| --- | --- | --- | --- |
| channel | $\hat{\beta}$ | *t* | *P* |
| Fp1 | 0.496 | 16.139 | <0.001 |
| Fp2 | 0.431 | 17.857 | <0.001 |
| F3 | 0.412 | 15.010 | <0.001 |
| F4 | 0.478 | 17.524 | <0.001 |
| C3 | 0.358 | 15.642 | <0.001 |
| C4 | 0.472 | 17.009 | <0.001 |
| P3 | 0.444 | 17.849 | <0.001 |
| P4 | 0.509 | 17.539 | <0.001 |
| O1 | 0.385 | 16.498 | <0.001 |
| O2 | 0.415 | 16.159 | <0.001 |
| F7 | 0.529 | 17.125 | <0.001 |
| F8 | 0.444 | 17.115 | <0.001 |
| T3 | 0.603 | 17.571 | <0.001 |
| T4 | 0.504 | 17.371 | <0.001 |
| T5 | 0.499 | 17.386 | <0.001 |
| T6 | 0.508 | 17.001 | <0.001 |

PSD: Power spectral density

| **Supplementary Table 5** The results of the interaction effect test in the  linear mixed model for the PSD Ratio5 feature set | | | |
| --- | --- | --- | --- |
| channel | $\hat{\beta}$ | *t* | *P* |
| Fp1 | 1.236 | 15.095 | <0.001 |
| Fp2 | 1.252 | 15.207 | <0.001 |
| F3 | 0.979 | 15.470 | <0.001 |
| F4 | 0.980 | 16.008 | <0.001 |
| C3 | 0.937 | 15.650 | <0.001 |
| C4 | 1.034 | 15.815 | <0.001 |
| P3 | 1.006 | 15.719 | <0.001 |
| P4 | 1.065 | 15.893 | <0.001 |
| O1 | 0.835 | 14.976 | <0.001 |
| O2 | 0.898 | 15.308 | <0.001 |
| F7 | 1.289 | 16.111 | <0.001 |
| F8 | 1.387 | 15.970 | <0.001 |
| T3 | 1.317 | 17.795 | <0.001 |
| T4 | 1.218 | 17.520 | <0.001 |
| T5 | 1.024 | 16.382 | <0.001 |
| T6 | 1.256 | 16.190 | <0.001 |

PSD: Power spectral density

| **Supplementary Table 6** The results of the interaction effect test in the  linear mixed model for the PSDE (deltaband) feature set | | | |
| --- | --- | --- | --- |
| channel | $\hat{\beta}$ | *t* | *P* |
| Fp1 | 0.004 | 7.487 | <0.001 |
| Fp2 | 0.005 | 7.467 | <0.001 |
| F3 | 0.004 | 7.084 | <0.001 |
| F4 | 0.004 | 7.957 | <0.001 |
| C3 | 0.004 | 7.612 | <0.001 |
| C4 | 0.004 | 8.089 | <0.001 |
| P3 | 0.004 | 7.602 | <0.001 |
| P4 | 0.004 | 7.903 | <0.001 |
| O1 | 0.004 | 7.796 | <0.001 |
| O2 | 0.004 | 7.900 | <0.001 |
| F7 | 0.004 | 7.629 | <0.001 |
| F8 | 0.004 | 7.735 | <0.001 |
| T3 | 0.004 | 6.960 | <0.001 |
| T4 | 0.004 | 7.439 | <0.001 |
| T5 | 0.004 | 7.897 | <0.001 |
| T6 | 0.004 | 7.970 | <0.001 |

PSDE: Power spectral density entropy

| **Supplementary Table 7** The results of the interaction effect test in the  linear mixed model for the PSDE (thetaband) feature set | | | |
| --- | --- | --- | --- |
| channel | $\hat{\beta}$ | *t* | *P* |
| Fp1 | 0.004 | 5.140 | <0.001 |
| Fp2 | 0.004 | 4.891 | <0.001 |
| F3 | 0.004 | 4.990 | <0.001 |
| F4 | 0.004 | 4.181 | <0.001 |
| C3 | 0.004 | 4.980 | <0.001 |
| C4 | 0.004 | 5.083 | <0.001 |
| P3 | 0.004 | 4.593 | <0.001 |
| P4 | 0.005 | 4.077 | <0.001 |
| O1 | 0.005 | 3.971 | <0.001 |
| O2 | 0.006 | 3.493 | <0.001 |
| F7 | 0.004 | 5.424 | <0.001 |
| F8 | 0.004 | 5.654 | <0.001 |
| T3 | 0.005 | 5.336 | <0.001 |
| T4 | 0.004 | 5.006 | <0.001 |
| T5 | 0.005 | 4.301 | <0.001 |
| T6 | 0.005 | 4.038 | <0.001 |

PSDE: Power spectral density entropy

| **Supplementary Table 8** The results of the interaction effect test in the  linear mixed model for the PSDE (alphaband) feature set | | | |
| --- | --- | --- | --- |
| channel | $\hat{\beta}$ | *t* | *P* |
| Fp1 | -0.021 | -14.539 | <0.001 |
| Fp2 | -0.019 | -13.825 | <0.001 |
| F3 | -0.019 | -13.479 | <0.001 |
| F4 | -0.019 | -14.412 | <0.001 |
| C3 | -0.018 | -16.014 | <0.001 |
| C4 | -0.017 | -16.648 | <0.001 |
| P3 | -0.018 | -15.109 | <0.001 |
| P4 | -0.019 | -14.850 | <0.001 |
| O1 | -0.019 | -12.766 | <0.001 |
| O2 | -0.017 | -13.413 | <0.001 |
| F7 | -0.017 | -16.330 | <0.001 |
| F8 | -0.016 | -15.758 | <0.001 |
| T3 | -0.015 | -17.120 | <0.001 |
| T4 | -0.015 | -18.754 | <0.001 |
| T5 | -0.021 | -16.005 | <0.001 |
| T6 | -0.018 | -14.714 | <0.001 |

PSDE: Power spectral density entropy

| **Supplementary Table 9** The results of the interaction effect test in the  linear mixed model for the PSDE (betaband) feature set | | | |
| --- | --- | --- | --- |
| channel | $\hat{\beta}$ | *t* | *P* |
| Fp1 | -0.010 | -15.739 | <0.001 |
| Fp2 | -0.010 | -16.218 | <0.001 |
| F3 | -0.010 | -16.147 | <0.001 |
| F4 | -0.010 | -16.259 | <0.001 |
| C3 | -0.009 | -15.234 | <0.001 |
| C4 | -0.010 | -17.521 | <0.001 |
| P3 | -0.011 | -16.305 | <0.001 |
| P4 | -0.010 | -14.928 | <0.001 |
| O1 | -0.012 | -18.101 | <0.001 |
| O2 | -0.012 | -17.952 | <0.001 |
| F7 | -0.008 | -15.489 | <0.001 |
| F8 | -0.009 | -15.649 | <0.001 |
| T3 | -0.008 | -16.165 | <0.001 |
| T4 | -0.009 | -17.181 | <0.001 |
| T5 | -0.011 | -18.462 | <0.001 |
| T6 | -0.011 | -17.797 | <0.001 |

PSDE: Power spectral density entropy

| **Supplementary Table 10** The results of the interaction effect test in the  linear mixed model for the PSDE (gammaband) feature set | | | |
| --- | --- | --- | --- |
| channel | $\hat{\beta}$ | *t* | *P* |
| Fp1 | -0.010 | -15.464 | <0.001 |
| Fp2 | -0.009 | -15.346 | <0.001 |
| F3 | -0.008 | -15.900 | <0.001 |
| F4 | -0.009 | -15.979 | <0.001 |
| C3 | 0.004 | 6.023 | <0.001 |
| C4 | 0.004 | 6.117 | <0.001 |
| P3 | 0.004 | 6.301 | <0.001 |
| P4 | 0.004 | 5.665 | <0.001 |
| O1 | -0.007 | -15.087 | <0.001 |
| O2 | -0.008 | -15.325 | <0.001 |
| F7 | -0.006 | -14.074 | <0.001 |
| F8 | -0.006 | -14.272 | <0.001 |
| T3 | -0.005 | -12.534 | <0.001 |
| T4 | -0.006 | -12.733 | <0.001 |
| T5 | -0.006 | -14.042 | <0.001 |
| T6 | -0.008 | -15.240 | <0.001 |

PSDE: Power spectral density entropy

| **Supplementary Table 11** The results of the interaction effect test in the  linear mixed model for the PE feature set | | | |
| --- | --- | --- | --- |
| channel | $\hat{\beta}$ | *t* | *P* |
| Fp1 | -9.992 | -3.727 | <0.001 |
| Fp2 | -7.223 | -2.307 | 0.022 |
| F3 | -7.703 | -3.176 | 0.002 |
| F4 | -8.397 | -3.455 | <0.001 |
| C3 | -6.380 | -3.912 | <0.001 |
| C4 | -6.666 | -3.890 | <0.001 |
| P3 | -9.049 | -3.489 | <0.001 |
| P4 | -11.588 | -3.167 | 0.002 |
| O1 | -12.586 | -2.851 | 0.005 |
| O2 | -13.236 | -2.889 | 0.004 |
| F7 | -8.009 | -3.451 | <0.001 |
| F8 | -7.774 | -3.998 | <0.001 |
| T3 | -9.368 | -3.951 | <0.001 |
| T4 | -8.357 | -3.772 | <0.001 |
| T5 | -10.996 | -2.771 | 0.006 |
| T6 | -10.453 | -2.432 | 0.016 |

PE: Permutation entropy

| **Supplementary Table 12** The results of the interaction effect test in the  linear mixed model for the SE feature set | | | |
| --- | --- | --- | --- |
| channel | $\hat{\beta}$ | *t* | *P* |
| Fp1 | -0.094 | -11.750 | <0.001 |
| Fp2 | -0.086 | -10.144 | <0.001 |
| F3 | -0.085 | -11.355 | <0.001 |
| F4 | -0.092 | -10.642 | <0.001 |
| C3 | -0.086 | -11.313 | <0.001 |
| C4 | -0.102 | -11.397 | <0.001 |
| P3 | -0.088 | -11.768 | <0.001 |
| P4 | -0.085 | -12.820 | <0.001 |
| O1 | -0.081 | -7.457 | <0.001 |
| O2 | -0.092 | -10.019 | <0.001 |
| F7 | -0.097 | -12.152 | <0.001 |
| F8 | -0.099 | -11.206 | <0.001 |
| T3 | -0.125 | -12.147 | <0.001 |
| T4 | -0.119 | -13.575 | <0.001 |
| T5 | -0.092 | -11.430 | <0.001 |
| T6 | -0.098 | -11.722 | <0.001 |

SE: Sample entropy

| **Supplementary Table 13** The results of the interaction effect test in the  linear mixed model for the M-DCPSR feature set | | | |
| --- | --- | --- | --- |
| channel | $\hat{\beta}$ | *t* | *P* |
| Fp1 | -0.032 | -13.474 | <0.001 |
| Fp2 | -0.030 | -13.504 | <0.001 |
| F3 | -0.030 | -12.227 | <0.001 |
| F4 | -0.029 | -11.404 | <0.001 |
| C3 | -0.029 | -13.096 | <0.001 |
| C4 | -0.029 | -13.010 | <0.001 |
| P3 | -0.030 | -13.136 | <0.001 |
| P4 | -0.033 | -14.345 | <0.001 |
| O1 | -0.036 | -15.685 | <0.001 |
| O2 | -0.036 | -15.605 | <0.001 |
| F7 | -0.028 | -14.610 | <0.001 |
| F8 | -0.028 | -14.995 | <0.001 |
| T3 | -0.030 | -17.628 | <0.001 |
| T4 | -0.028 | -17.009 | <0.001 |
| T5 | -0.031 | -15.435 | <0.001 |
| T6 | -0.034 | -17.264 | <0.001 |

M-DCPSR: Median distance from the centroid of phase space reconstruction

**Supplementary Table 14** The results of the interaction effect test in the linear mixed model for the PLI feature set

| channel pairs | $\hat{\beta}$ | *t* | *P* | channel pairs | $\hat{\beta}$ | *t* | *P* |
| --- | --- | --- | --- | --- | --- | --- | --- |
| Fp1 - Fp2 | -0.041 | -3.962 | <0.001 | C3 - F8 | -0.036 | -5.175 | <0.001 |
| Fp1 - F3 | -0.029 | -3.476 | <0.001 | C3 - T3 | -0.030 | -5.308 | <0.001 |
| Fp1 - F4 | -0.035 | -4.857 | <0.001 | C3 - T4 | -0.032 | -5.828 | <0.001 |
| Fp1 - C3 | -0.044 | -5.423 | <0.001 | C3 - T5 | -0.036 | -5.223 | <0.001 |
| Fp1 - C4 | -0.045 | -5.531 | <0.001 | C3 - T6 | -0.036 | -5.343 | <0.001 |
| Fp1 - P3 | -0.045 | -4.880 | <0.001 | C4 - P3 | -0.036 | -5.062 | <0.001 |
| Fp1 - P4 | -0.042 | -5.638 | <0.001 | C4 - P4 | -0.049 | -5.871 | <0.001 |
| Fp1 - O1 | -0.038 | -5.466 | <0.001 | C4 - O1 | -0.040 | -4.681 | <0.001 |
| Fp1 - O2 | -0.044 | -4.838 | <0.001 | C4 - O2 | -0.046 | -5.586 | <0.001 |
| Fp1 - F7 | -0.037 | -5.353 | <0.001 | C4 - F7 | -0.040 | -5.720 | <0.001 |
| Fp1 - F8 | -0.030 | -5.343 | <0.001 | C4 - F8 | -0.040 | -6.115 | <0.001 |
| Fp1 - T3 | -0.034 | -5.039 | <0.001 | C4 - T3 | -0.034 | -5.544 | <0.001 |
| Fp1 - T4 | -0.040 | -4.821 | <0.001 | C4 - T4 | -0.028 | -5.030 | <0.001 |
| Fp1 - T5 | -0.040 | -5.631 | <0.001 | C4 - T5 | -0.033 | -5.374 | <0.001 |
| Fp1 - T6 | -0.051 | -4.864 | <0.001 | C4 - T6 | -0.043 | -5.275 | <0.001 |
| Fp2 - F3 | -0.044 | -4.085 | <0.001 | P3 - P4 | -0.037 | -5.305 | <0.001 |
| Fp2 - F4 | -0.045 | -5.271 | <0.001 | P3 - O1 | -0.056 | -6.792 | <0.001 |
| Fp2 - C3 | -0.044 | -5.003 | <0.001 | P3 - O2 | -0.046 | -5.363 | <0.001 |
| Fp2 - C4 | -0.048 | -6.127 | <0.001 | P3 - F7 | -0.036 | -5.477 | <0.001 |
| Fp2 - P3 | -0.044 | -5.364 | <0.001 | P3 - F8 | -0.032 | -5.453 | <0.001 |
| Fp2 - P4 | -0.039 | -5.161 | <0.001 | P3 - T3 | -0.032 | -5.672 | <0.001 |
| Fp2 - O1 | -0.045 | -6.049 | <0.001 | P3 - T4 | -0.032 | -4.750 | <0.001 |
| Fp2 - O2 | -0.048 | -5.443 | <0.001 | P3 - T5 | -0.058 | -6.462 | <0.001 |
| Fp2 - F7 | -0.032 | -4.780 | <0.001 | P3 - T6 | -0.040 | -4.793 | <0.001 |
| Fp2 - F8 | -0.034 | -5.962 | <0.001 | P4 - O1 | -0.041 | -5.023 | <0.001 |
| Fp2 - T3 | -0.033 | -5.607 | <0.001 | P4 - O2 | -0.057 | -6.929 | <0.001 |
| Fp2 - T4 | -0.038 | -4.740 | <0.001 | P4 - F7 | -0.033 | -4.849 | <0.001 |
| Fp2 - T5 | -0.050 | -5.653 | <0.001 | P4 - F8 | -0.029 | -5.637 | <0.001 |
| Fp2 - T6 | -0.046 | -4.450 | <0.001 | P4 - T3 | -0.034 | -4.673 | <0.001 |
| F3 - F4 | -0.028 | -5.238 | <0.001 | P4 - T4 | -0.039 | -5.388 | <0.001 |
| F3 - C3 | -0.045 | -5.552 | <0.001 | P4 - T5 | -0.044 | -5.538 | <0.001 |
| F3 - C4 | -0.039 | -4.619 | <0.001 | P4 - T6 | -0.055 | -5.972 | <0.001 |
| F3 - P3 | -0.048 | -5.493 | <0.001 | O1 - O2 | -0.040 | -5.609 | <0.001 |
| F3 - P4 | -0.044 | -5.338 | <0.001 | O1 - F7 | -0.039 | -5.601 | <0.001 |
| F3 - O1 | -0.039 | -5.179 | <0.001 | O1 - F8 | -0.036 | -5.340 | <0.001 |
| F3 - O2 | -0.041 | -5.661 | <0.001 | O1 - T3 | -0.035 | -4.877 | <0.001 |
| F3 - F7 | -0.039 | -5.040 | <0.001 | O1 - T4 | -0.034 | -4.828 | <0.001 |
| F3 - F8 | -0.029 | -5.141 | <0.001 | O1 - T5 | -0.050 | -5.217 | <0.001 |
| F3 - T3 | -0.035 | -5.124 | <0.001 | O1 - T6 | -0.047 | -4.656 | <0.001 |
| F3 - T4 | -0.036 | -4.991 | <0.001 | O2 - F7 | -0.044 | -6.393 | <0.001 |
| F3 - T5 | -0.037 | -5.359 | <0.001 | O2 - F8 | -0.035 | -5.082 | <0.001 |
| F3 - T6 | -0.045 | -4.630 | <0.001 | O2 - T3 | -0.036 | -4.871 | <0.001 |
| F4 - C3 | -0.039 | -4.337 | <0.001 | O2 - T4 | -0.038 | -5.251 | <0.001 |
| F4 - C4 | -0.047 | -5.759 | <0.001 | O2 - T5 | -0.040 | -5.210 | <0.001 |
| F4 - P3 | -0.048 | -5.562 | <0.001 | O2 - T6 | -0.050 | -4.873 | <0.001 |
| F4 - P4 | -0.045 | -5.929 | <0.001 | F7 - F8 | -0.024 | -5.366 | <0.001 |
| F4 - O1 | -0.036 | -5.321 | <0.001 | F7 - T3 | -0.030 | -5.228 | <0.001 |
| F4 - O2 | -0.033 | -5.174 | <0.001 | F7 - T4 | -0.035 | -5.780 | <0.001 |
| F4 - F7 | -0.038 | -5.687 | <0.001 | F7 - T5 | -0.038 | -5.783 | <0.001 |
| F4 - F8 | -0.039 | -6.037 | <0.001 | F7 - T6 | -0.046 | -6.026 | <0.001 |
| F4 - T3 | -0.034 | -5.354 | <0.001 | F8 - T3 | -0.030 | -5.790 | <0.001 |
| F4 - T4 | -0.037 | -5.337 | <0.001 | F8 - T4 | -0.035 | -6.781 | <0.001 |
| F4 - T5 | -0.043 | -5.210 | <0.001 | F8 - T5 | -0.040 | -6.178 | <0.001 |
| F4 - T6 | -0.042 | -4.533 | <0.001 | F8 - T6 | -0.036 | -5.233 | <0.001 |
| C3 - C4 | -0.033 | -5.935 | <0.001 | T3 - T4 | -0.028 | -5.574 | <0.001 |
| C3 - P3 | -0.047 | -5.770 | <0.001 | T3 - T5 | -0.033 | -5.951 | <0.001 |
| C3 - P4 | -0.041 | -4.979 | <0.001 | T3 - T6 | -0.038 | -4.676 | <0.001 |
| C3 - O1 | -0.045 | -6.092 | <0.001 | T4 - T5 | -0.034 | -5.461 | <0.001 |
| C3 - O2 | -0.041 | -4.782 | <0.001 | T4 - T6 | -0.037 | -5.307 | <0.001 |
| C3 - F7 | -0.041 | -5.617 | <0.001 | T5 - T6 | -0.039 | -5.171 | <0.001 |

PLI: Phase lag index
