# Supplementary material for: Predicting progression from amnestic mild cognitive impairment to Alzheimer's disease using longitudinal EEG data: a 12-month cohort study
Source: Front Aging Neurosci. 2026 Jan 20;17:1719981. doi: 10.3389/fnagi.2025.1719981 (PMC12864486; doi:10.3389/fnagi.2025.1719981)
Supplement: Supplementary file 2 [file Image_1.pdf]

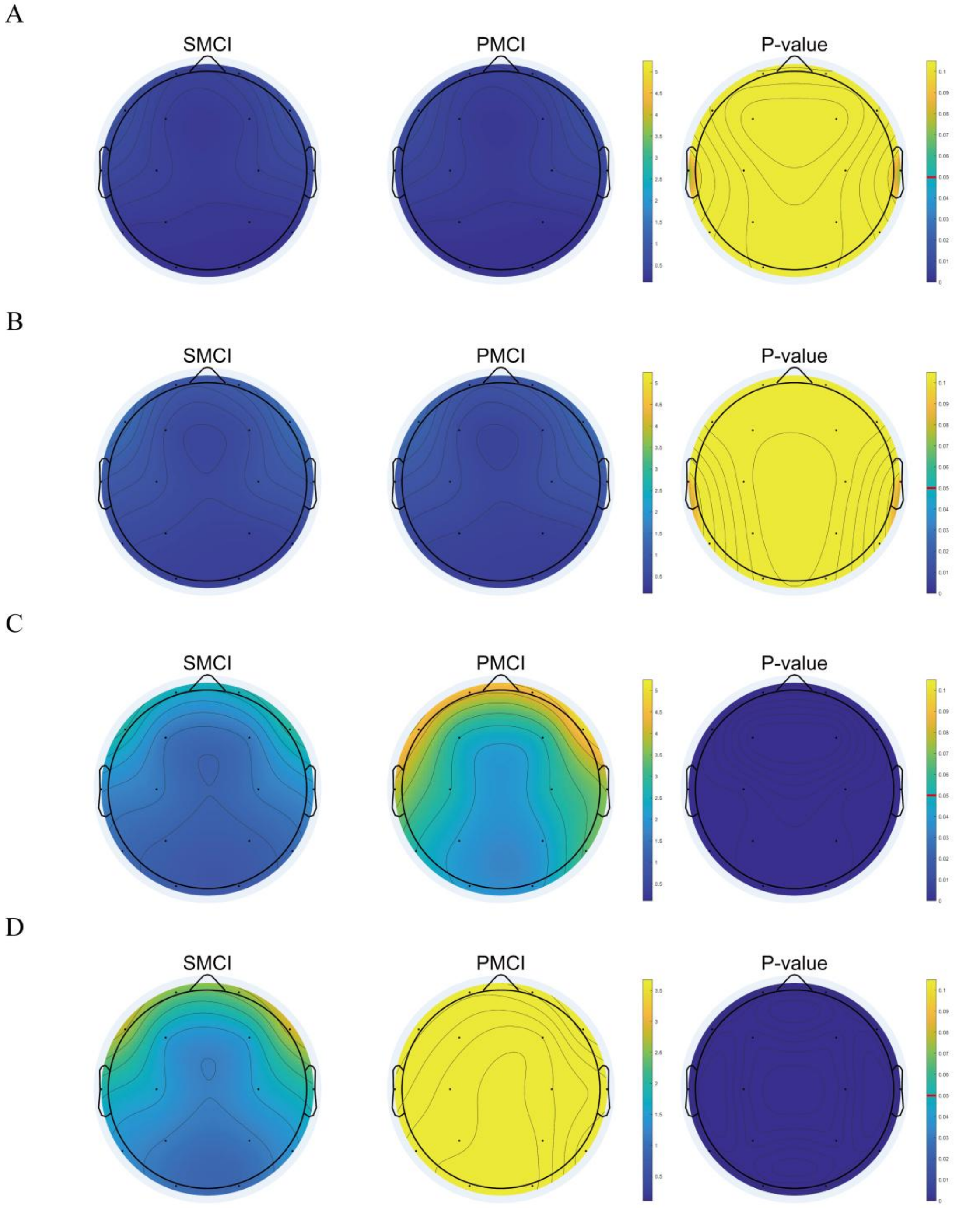

Supplementary Figure 1 The topoplot of original value and statistical differences for Power spectral density (PSD) Ratio1 in stable mild cognitive impairment (SMCI) and progressive mild cognitive impairment (PMCI) groups including 0 month (A), 4 months (B), 8 months (C), 12months (D). The first and second column of each sub-figure are the topoplots of mean in SMCI and PMCI groups. The third column of each sub-figure is the topoplot of statistical differences in SMCI and PMCI groups.

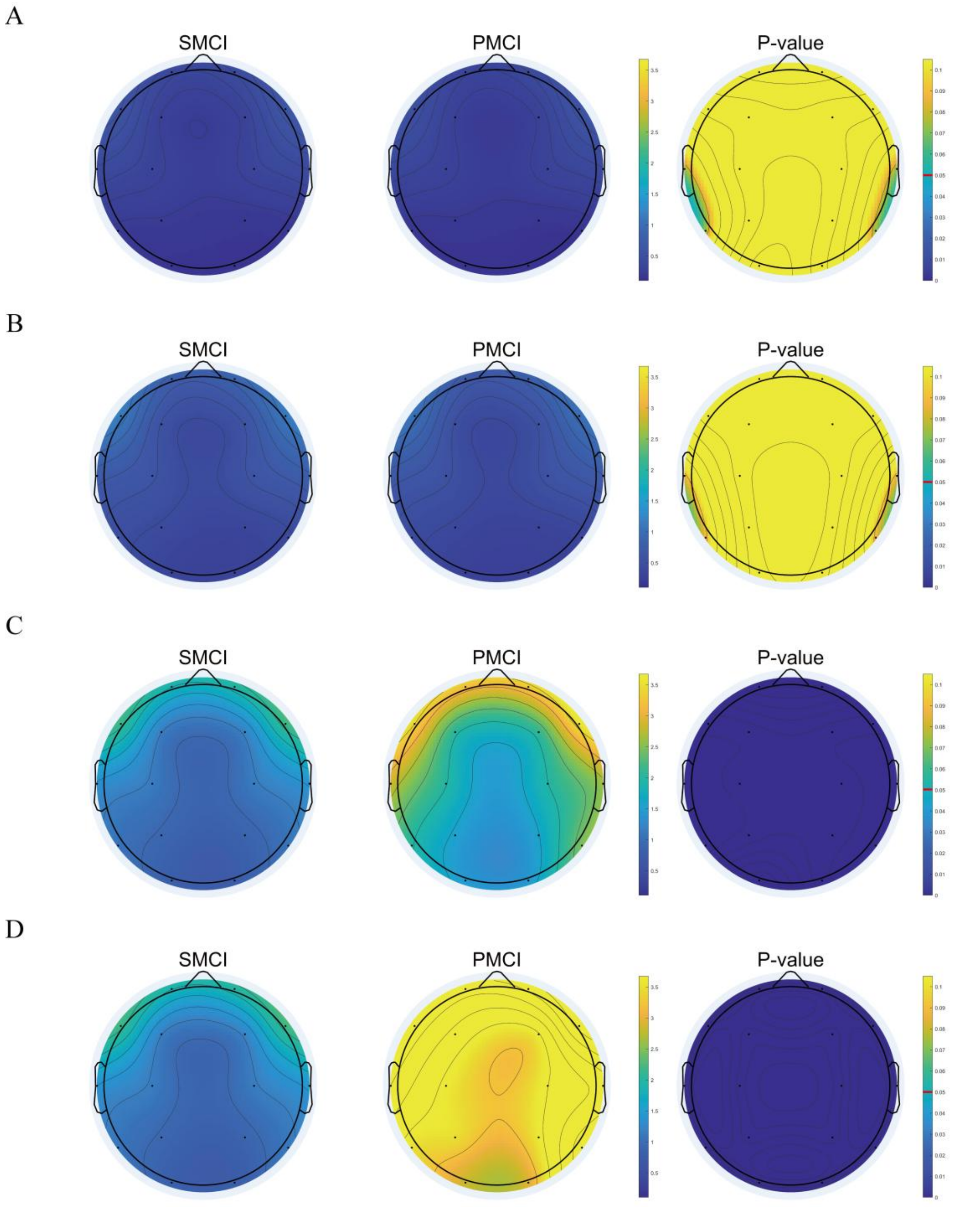

Supplementary Figure 2 The topoplot of original value and statistical differences for Power spectral density (PSD) Ratio3 in stable mild cognitive impairment (SMCI) and progressive mild cognitive impairment (PMCI) groups including 0 month (A), 4 months (B), 8 months (C), 12months (D). The first and second column of each sub-figure are the topoplots of mean in SMCI and PMCI groups. The third column of each sub-figure is the topoplot of statistical differences in SMCI and PMCI groups.

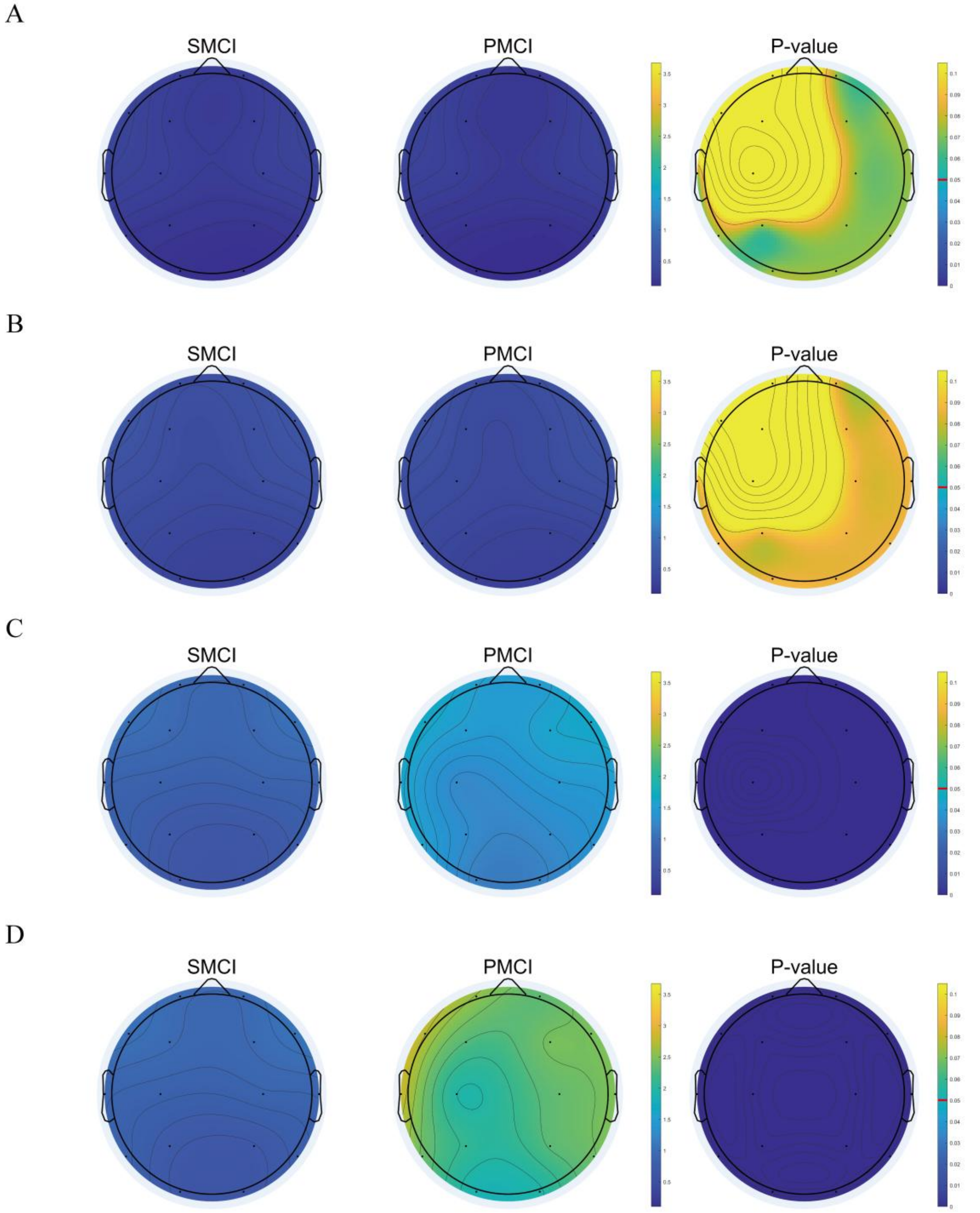

Supplementary Figure 3 The topoplot of original value and statistical differences for Power spectral density (PSD) Ratio4 in stable mild cognitive impairment (SMCI) and progressive mild cognitive impairment (PMCI) groups including 0 month (A), 4 months (B), 8 months (C), 12months (D). The first and second column of each sub-figure are the topoplots of mean in SMCI and PMCI groups. The third column of each sub-figure is the topoplot of statistical differences in SMCI and PMCI groups.

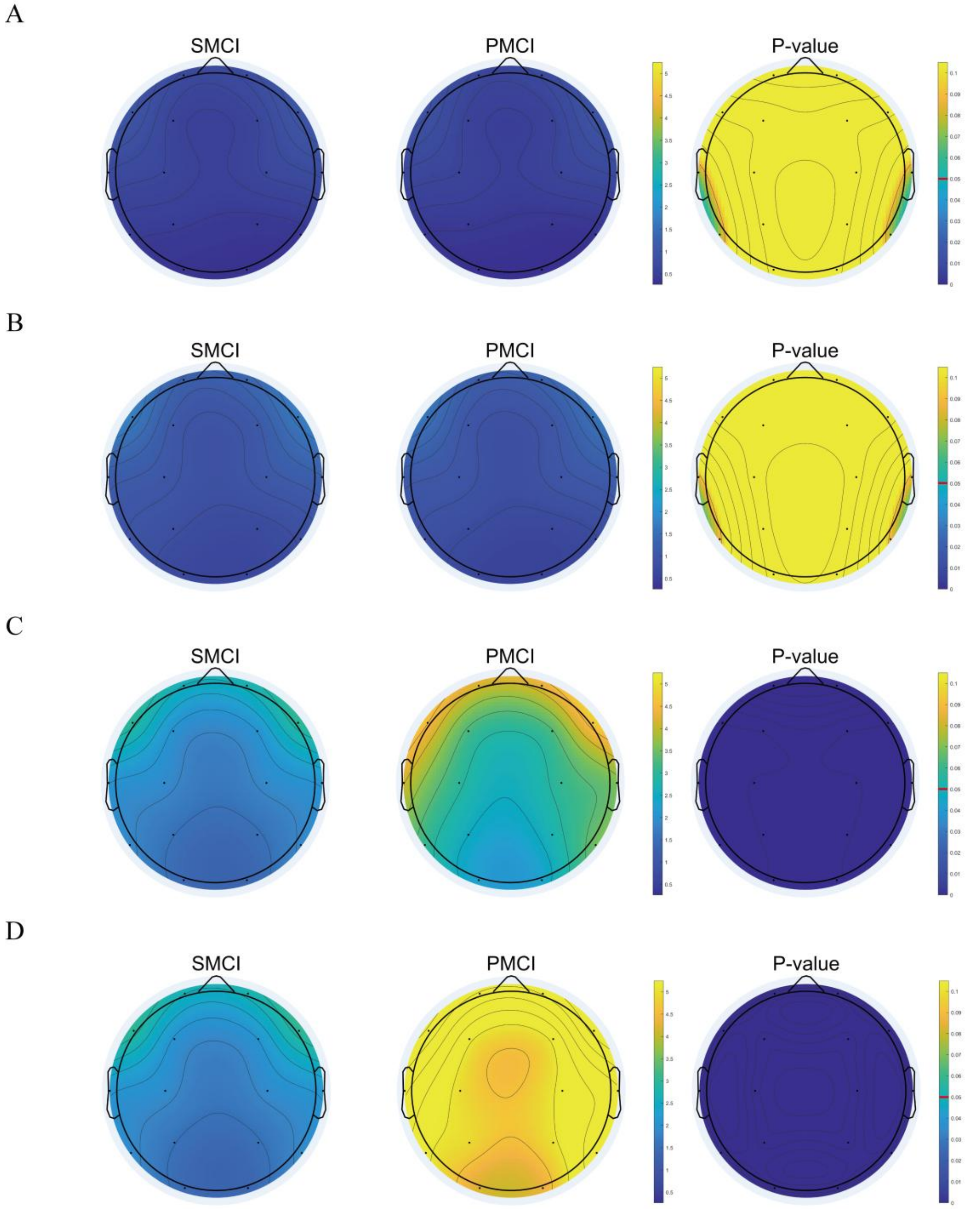

Supplementary Figure 4 The topoplot of original value and statistical differences for Power spectral density (PSD) Ratio5 in stable mild cognitive impairment (SMCI) and progressive mild cognitive impairment (PMCI) groups including 0 month (A), 4 months (B), 8 months (C), 12months (D). The first and second column of each sub-figure are the topoplots of mean in SMCI and PMCI groups. The third column of each sub-figure is the topoplot of statistical differences in SMCI and PMCI groups.

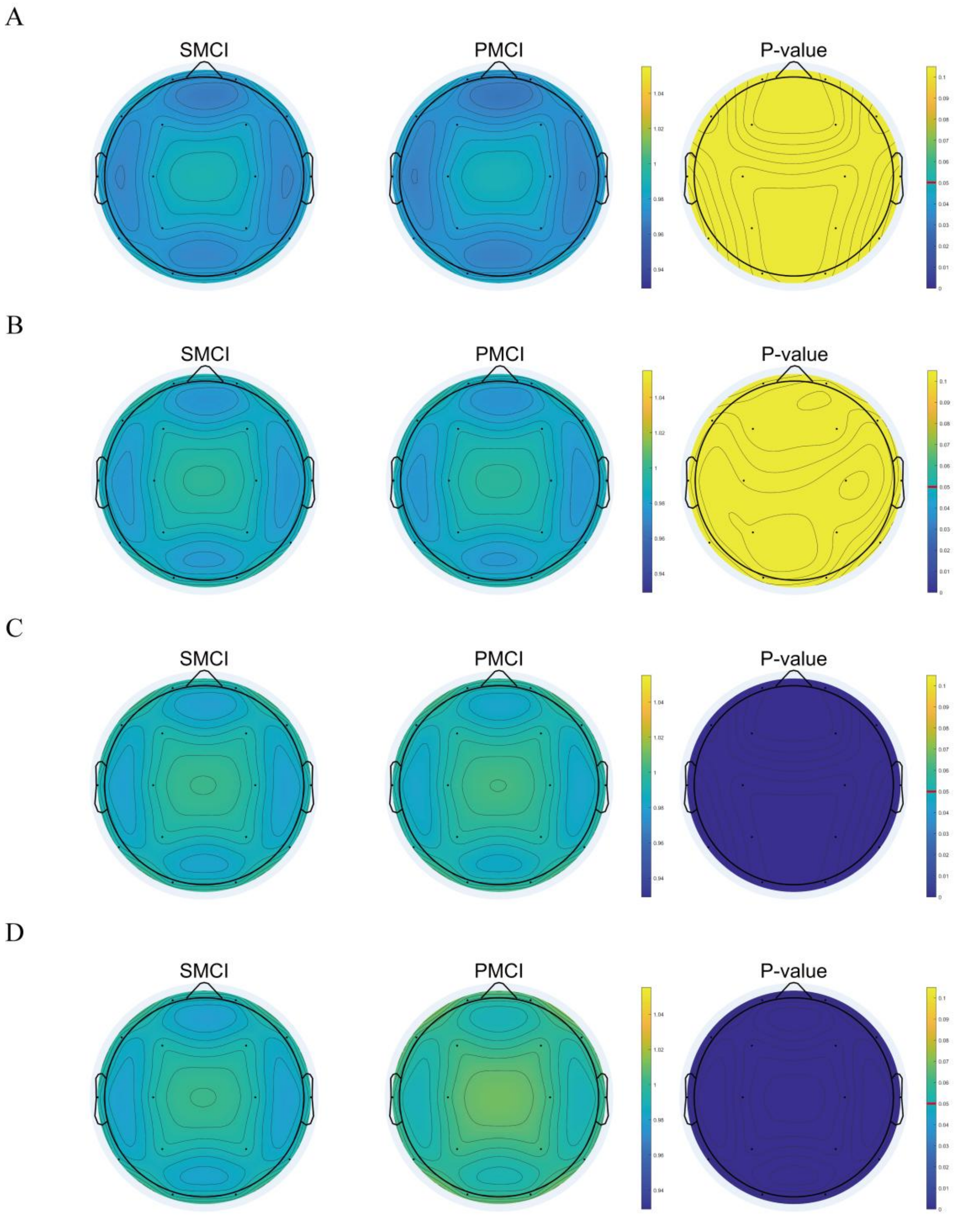

Supplementary Figure 5 The topoplot of original value and statistical differences for Power spectral density entropy (PSDE) in delta band in stable mild cognitive impairment (SMCI) and progressive mild cognitive impairment (PMCI) groups including 0 month (A), 4 months (B), 8 months (C), 12months (D). The first and second column of each sub-figure are the topoplots of mean in SMCI and PMCI groups. The third column of each sub-figure is the topoplot of statistical differences in SMCI and PMCI groups.

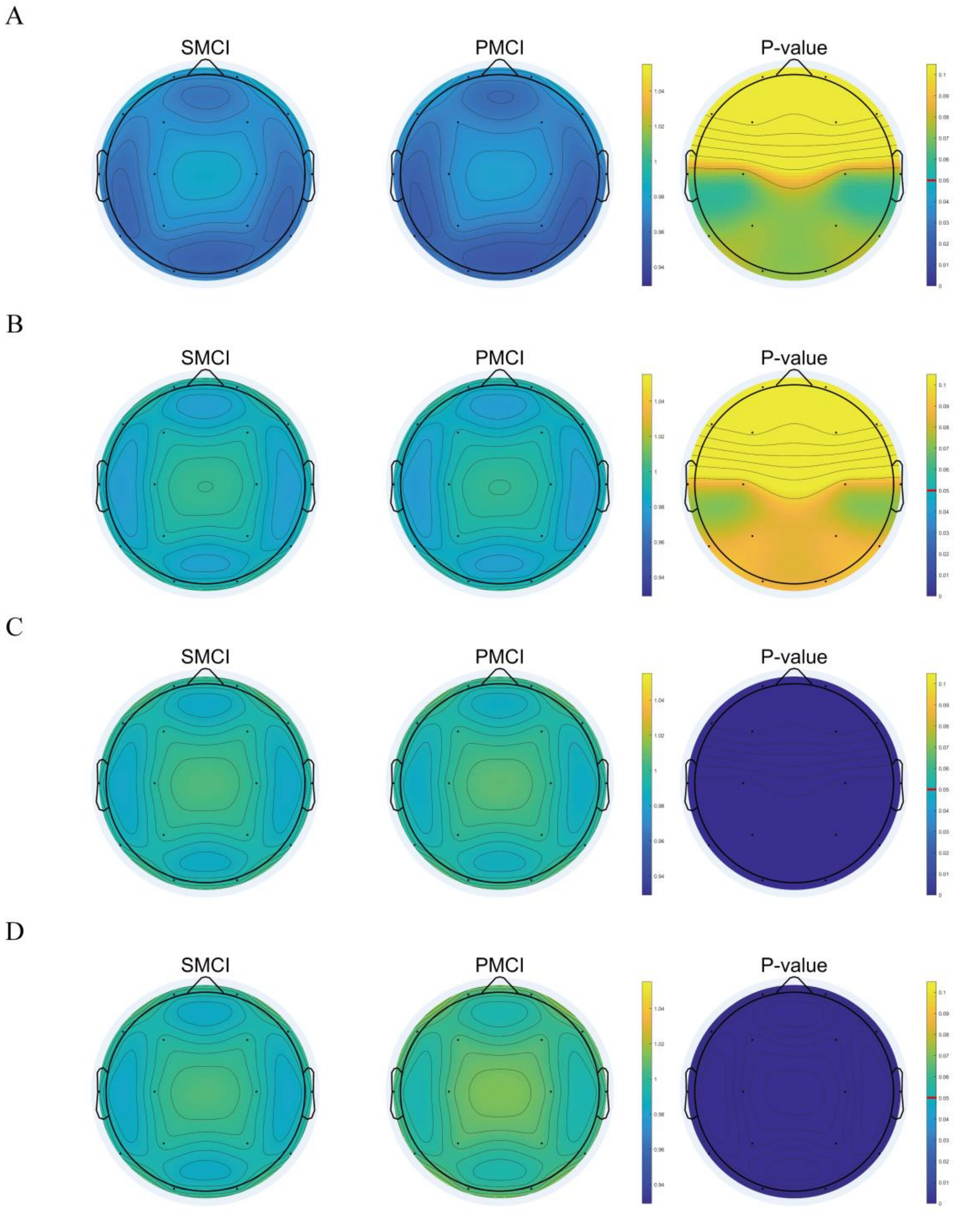

Supplementary Figure 6 The topoplot of original value and statistical differences for Power spectral density entropy (PSDE) in theta band in stable mild cognitive impairment (SMCI) and progressive mild cognitive impairment (PMCI) groups including 0 month (A), 4 months (B), 8 months (C), 12months (D). The first and second column of each sub-figure are the topoplots of mean in SMCI and PMCI groups. The third column of each sub-figure is the topoplot of statistical differences in SMCI and PMCI groups.

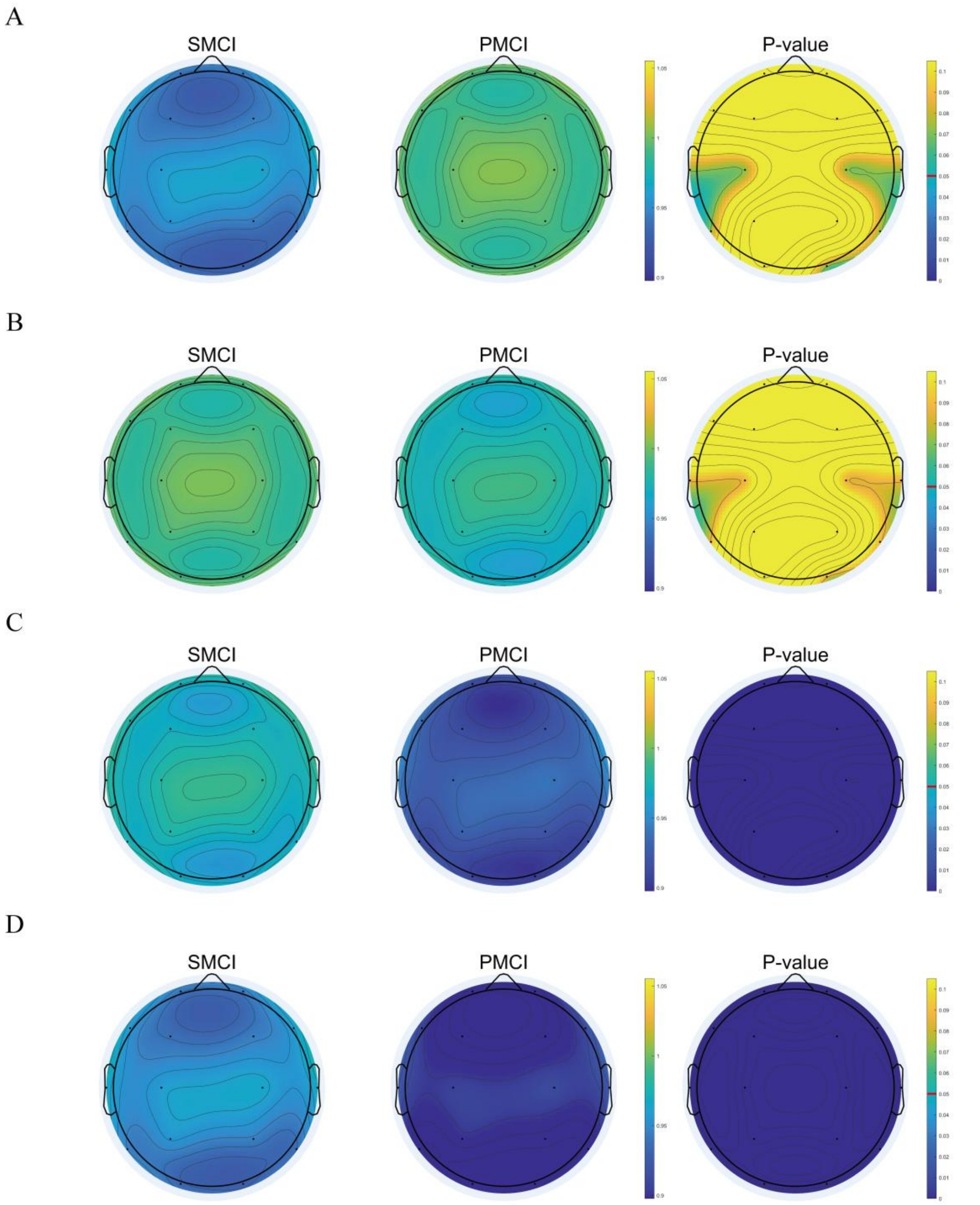

Supplementary Figure 7 The topoplot of original value and statistical differences for Power spectral density entropy (PSDE) in alpha band in stable mild cognitive impairment (SMCI) and progressive mild cognitive impairment (PMCI) groups including 0 month (A), 4 months (B), 8 months (C), 12months (D). The first and second column of each sub-figure are the topoplots of mean in SMCI and PMCI groups. The third column of each sub-figure is the topoplot of statistical differences in SMCI and PMCI groups.

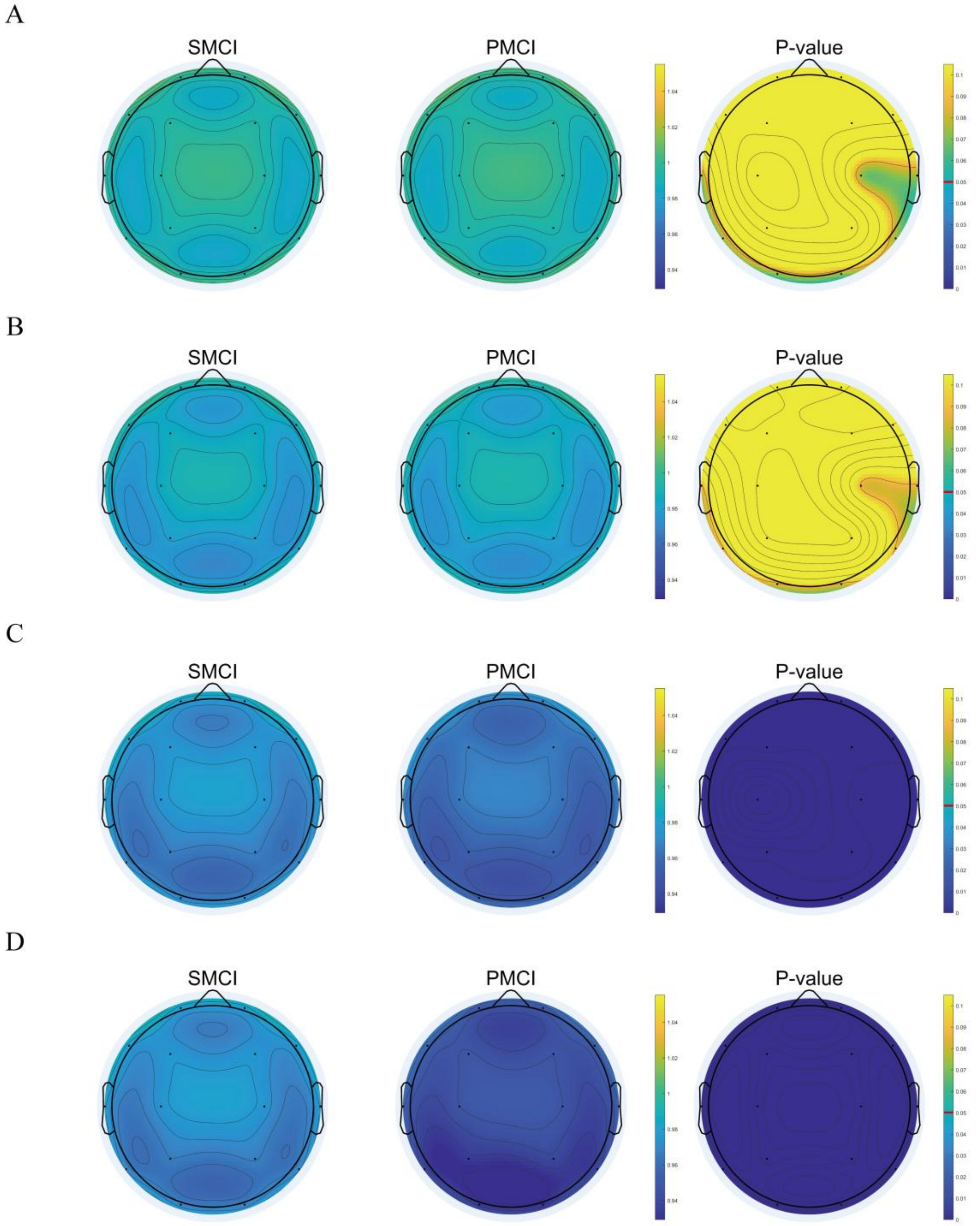

Supplementary Figure 8 The topoplot of original value and statistical differences for Power spectral density entropy (PSDE) in beta band in stable mild cognitive impairment (SMCI) and progressive mild cognitive impairment (PMCI) groups including 0 month (A), 4 months (B), 8 months (C), 12months (D). The first and second column of each sub-figure are the topoplots of mean in SMCI and PMCI groups. The third column of each sub-figure is the topoplot of statistical differences in SMCI and PMCI groups.

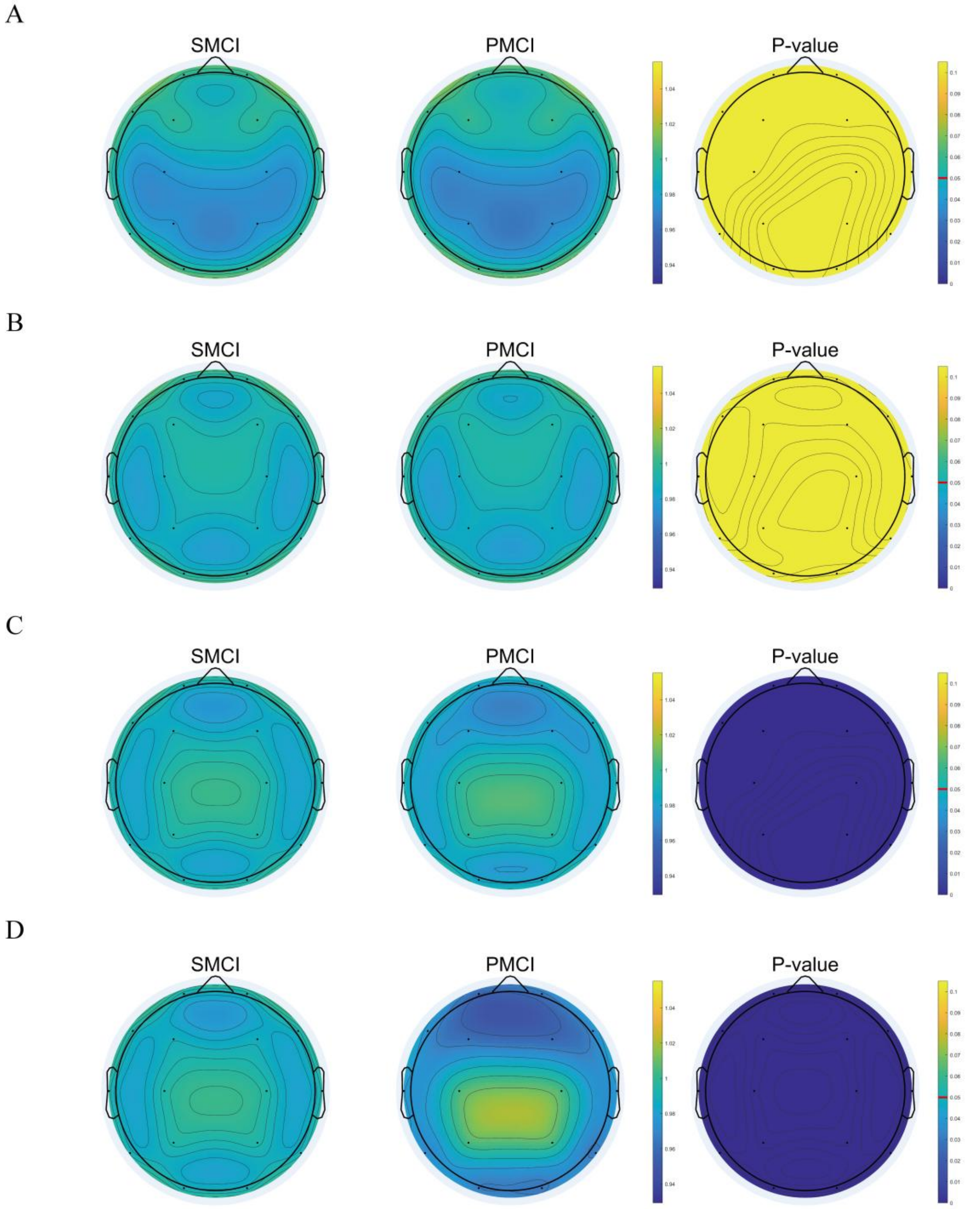

Supplementary Figure 9 The topoplot of original value and statistical differences for Power spectral density entropy (PSDE) in gamma band in stable mild cognitive impairment (SMCI) and progressive mild cognitive impairment (PMCI) groups including 0 month (A), 4 months (B), 8 months (C), 12months (D). The first and second column of each sub-figure are the topoplots of mean in SMCI and PMCI groups. The third column of each sub-figure is the topoplot of statistical differences in SMCI and PMCI groups.

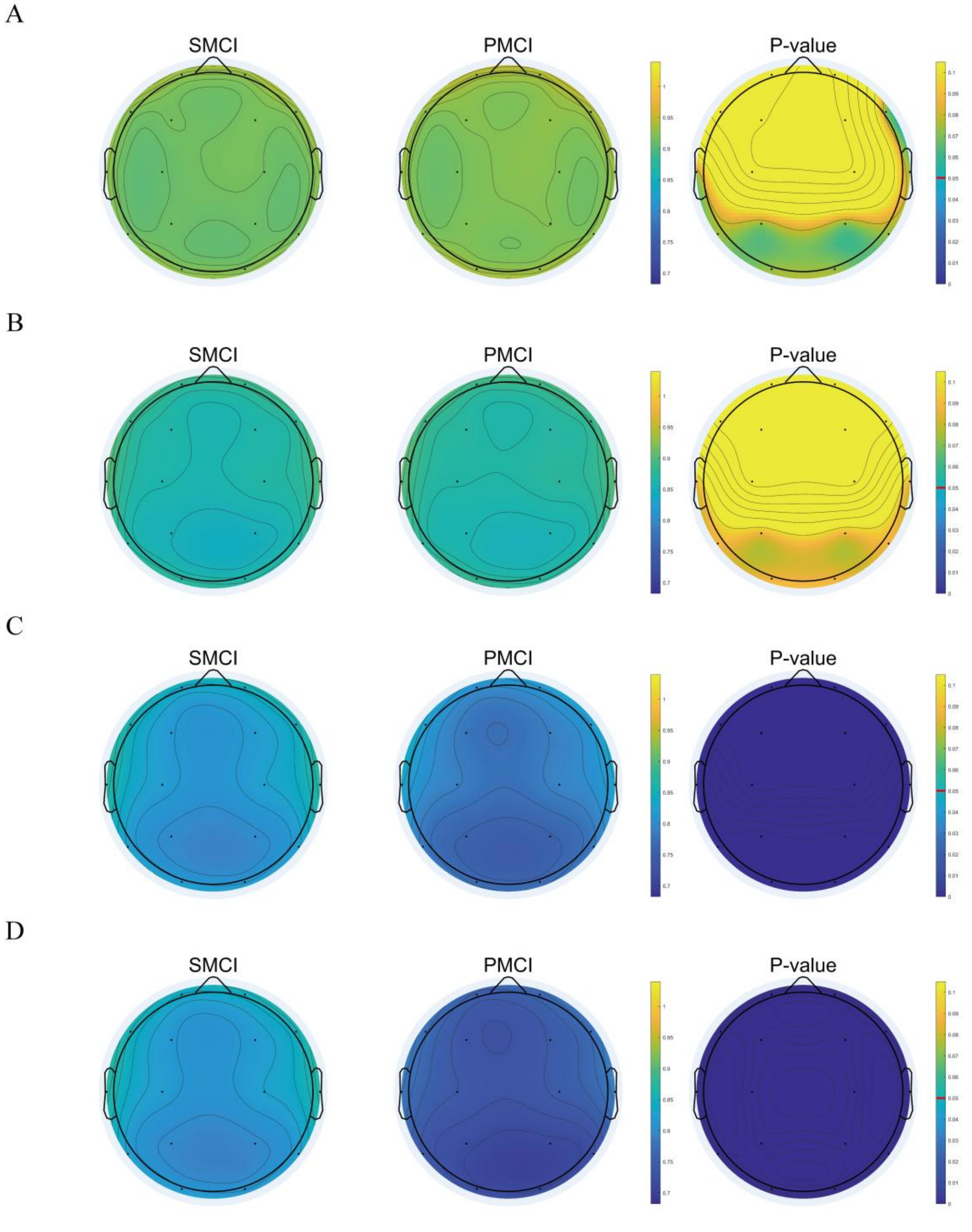

Supplementary Figure 10 The topoplot of original value and statistical differences for Permutation entropy (PE) in stable mild cognitive impairment (SMCI) and progressive mild cognitive impairment (PMCI) groups including 0 month (A), 4 months (B), 8 months (C), 12months (D). The first and second column of each sub-figure are the topoplots of mean in SMCI and PMCI groups. The third column of each sub-figure is the topoplot of statistical differences in SMCI and PMCI groups.

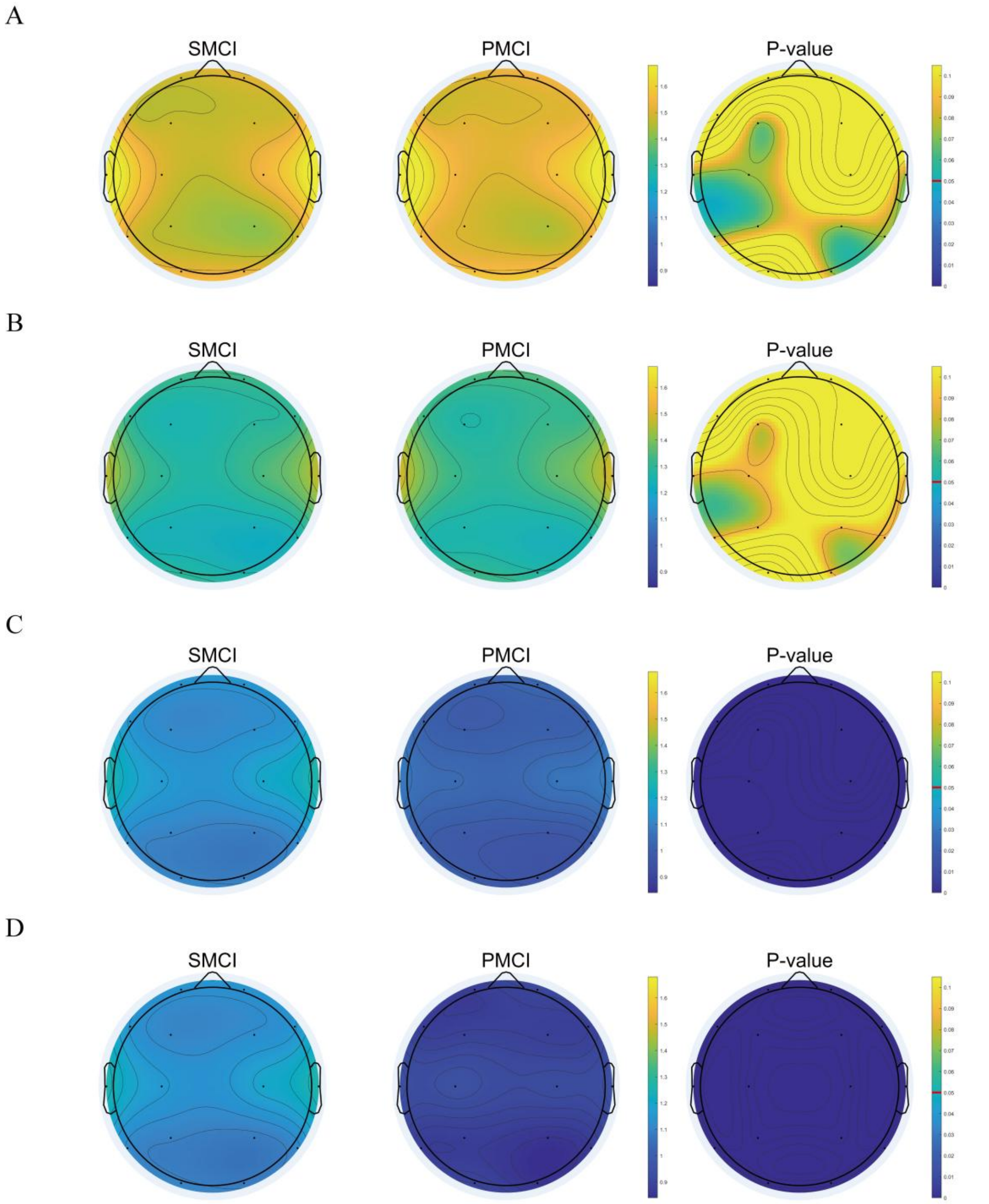

Supplementary Figure 11 The topoplot of original value and statistical differences for Sample entropy (SE) in stable mild cognitive impairment (SMCI) and progressive mild cognitive impairment (PMCI) groups including 0 month (A), 4 months (B), 8 months (C), 12months (D). The first and second column of each sub-figure are the topoplots of mean in SMCI and PMCI groups. The third column of each sub-figure is the topoplot of statistical differences in SMCI and PMCI groups.

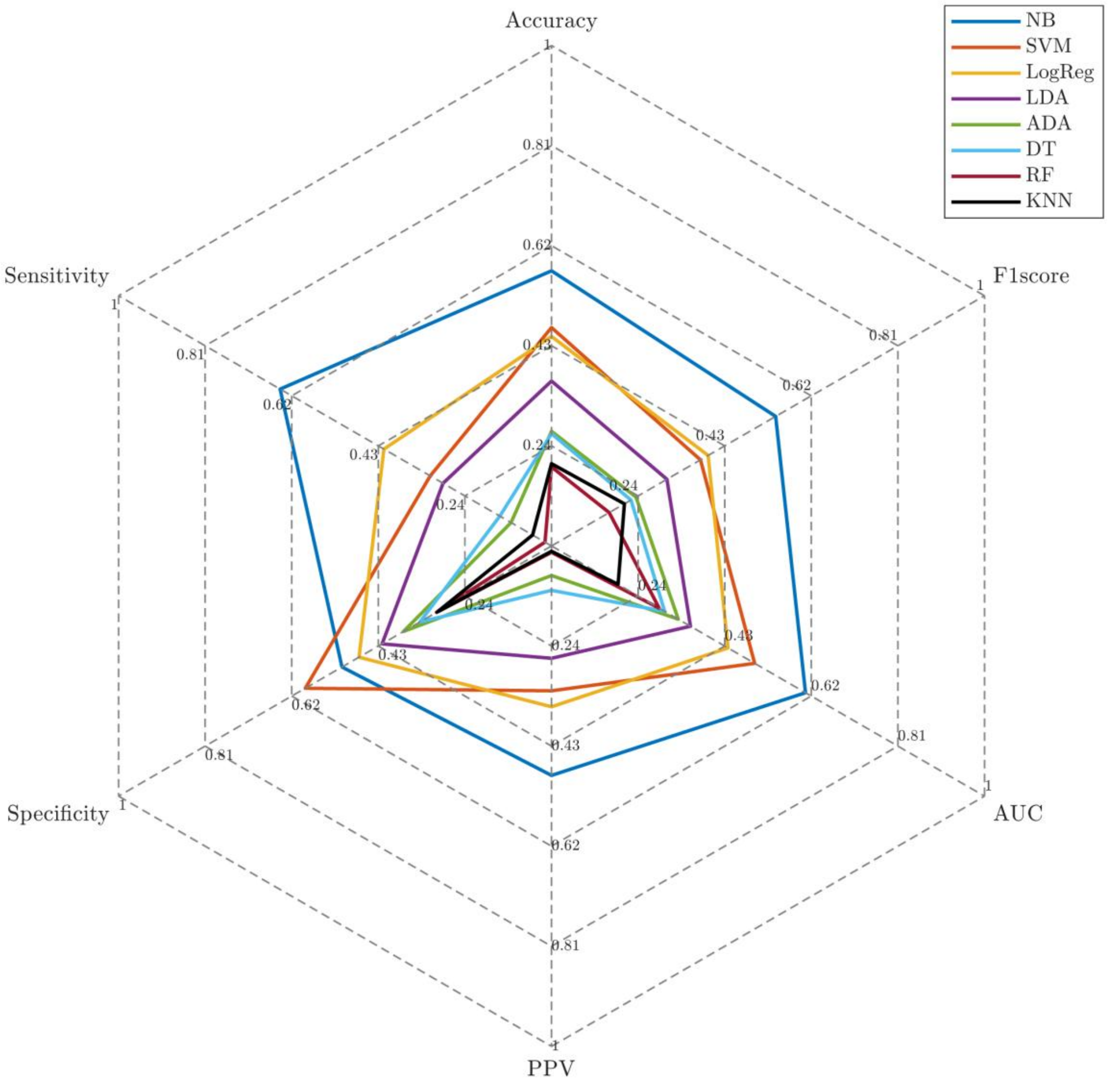

Supplementary Figure 12 The radar chart of prediction results using cross-sectional features of Time1 (repeated 10×5-fold cross validation).

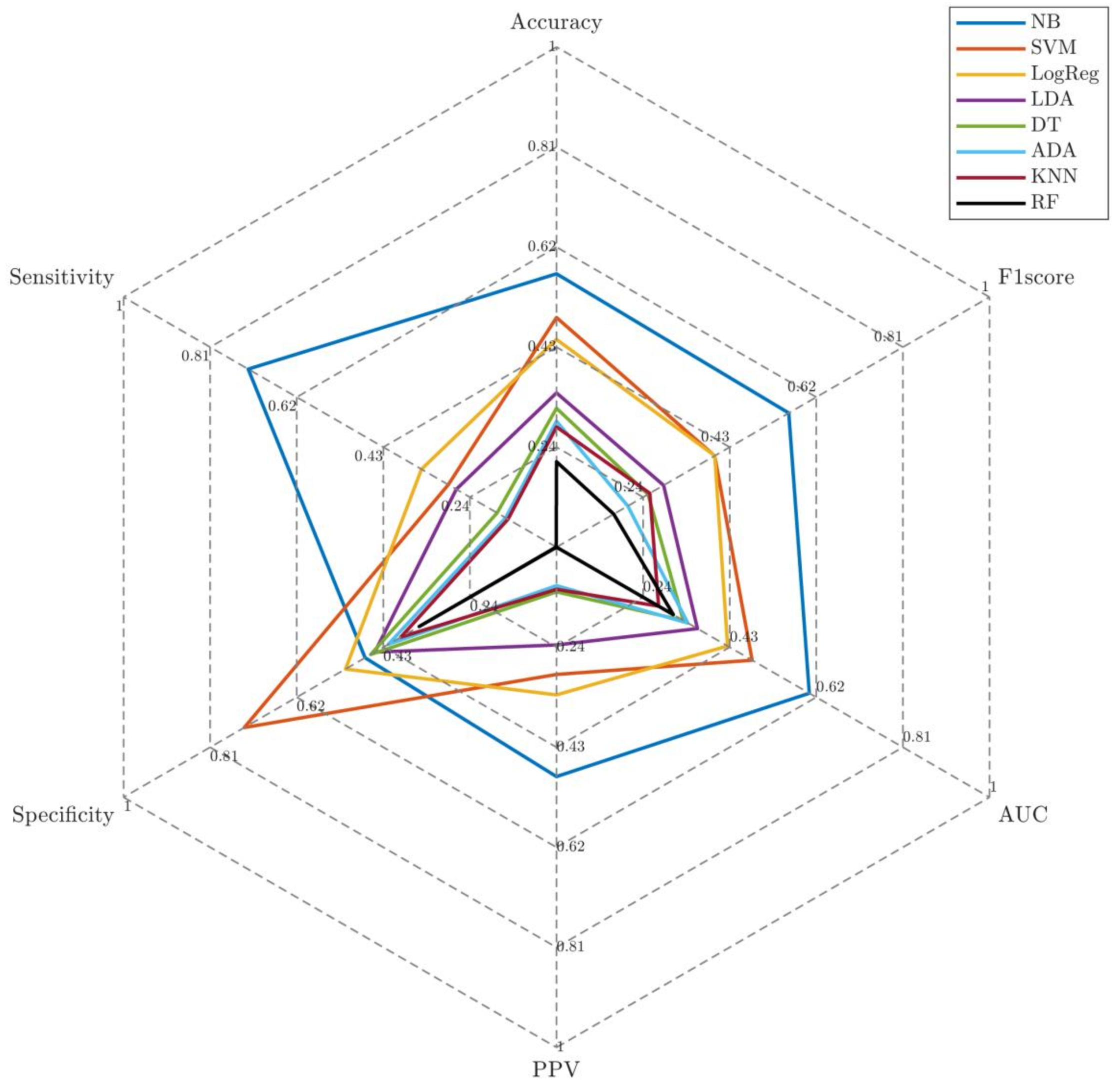

Supplementary Figure 13 The radar chart of prediction results using cross-sectional features of Time2 (repeated 10×5-fold cross validation).

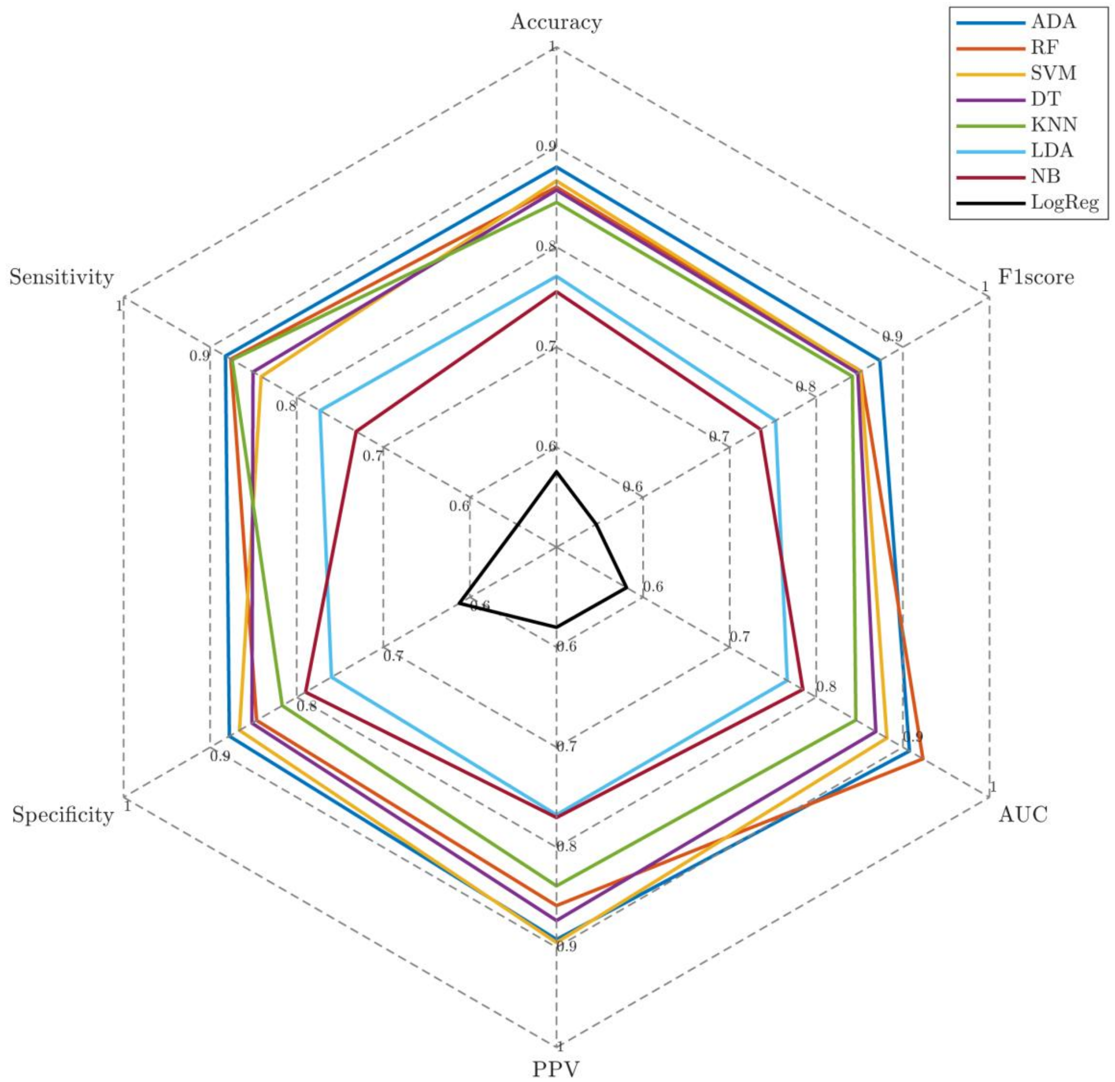

Supplementary Figure 14 The radar chart of prediction results using cross-sectional features of Time3 (repeated 10×5-fold cross validation).

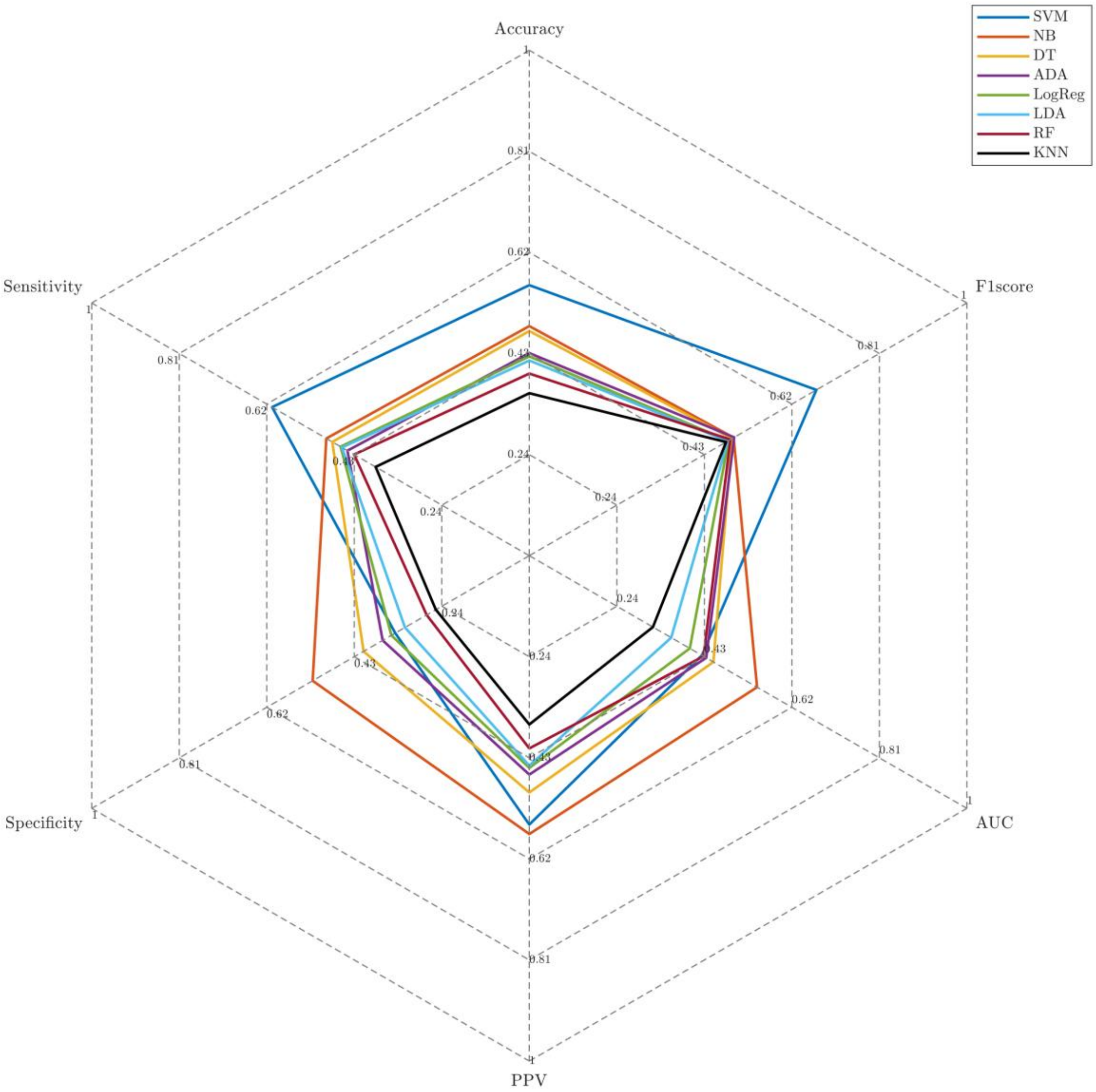

Supplementary Figure 15 The radar chart of prediction results using longitudinal features of Time1 and Time2 (repeated 10×5-fold cross validation).

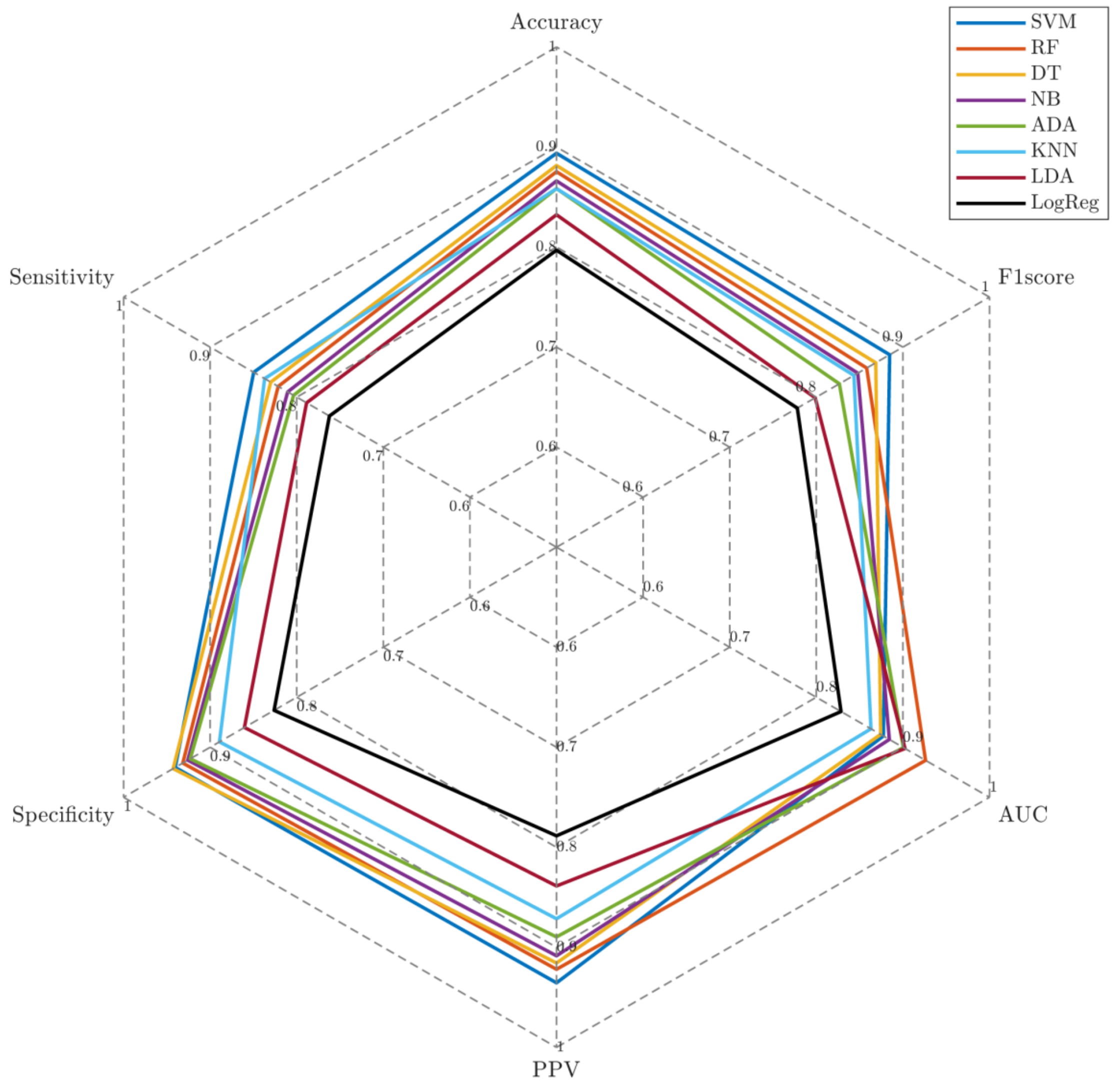

Supplementary Figure 16 The radar chart of prediction results using longitudinal features of Time1 and Time3 (repeated 10×5-fold cross validation).

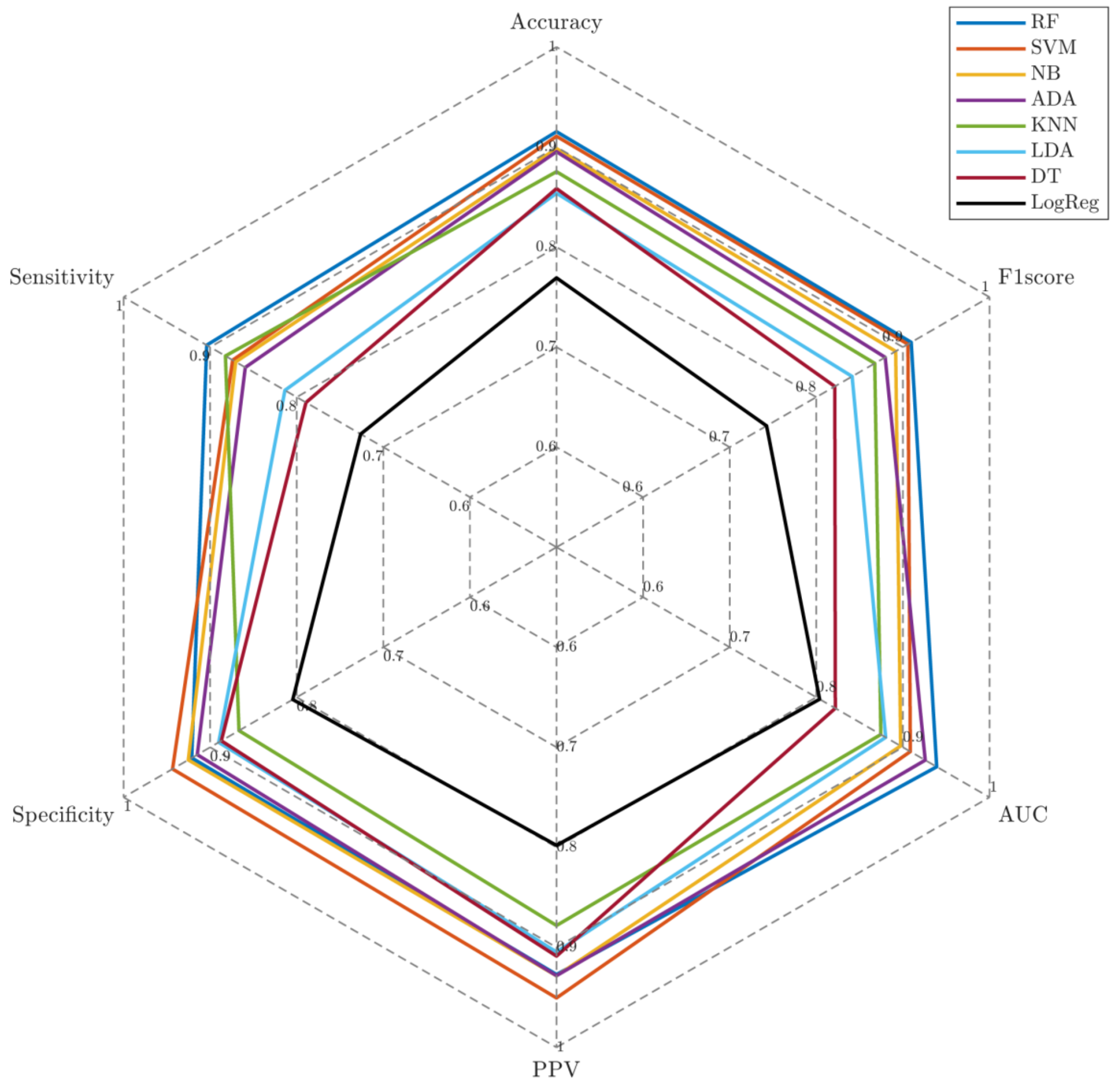

Supplementary Figure 17 The radar chart of prediction results using longitudinal features of Time2 and Time3 (repeated 10×5-fold cross validation).
